# *NF2* loss malignantly transforms human pancreatic acinar cells and enhances cell fitness under environmental stress

**DOI:** 10.1172/JCI194395

**Published:** 2026-01-02

**Authors:** Yi Xu, Michael H. Nipper, Angel A. Dominguez, Chenhui He, Francis E. Sharkey, Sajid Khan, Han Xu, Daohong Zhou, Lei Zheng, Yu Luan, Jun Liu, Pei Wang

**Affiliations:** 1 Department of Cell Systems and Anatomy,; 2 Department of Pathology and Laboratory Medicine, and; 3 Department of Biochemistry and Structural Biology, The University of Texas Health San Antonio, San Antonio, Texas, USA.; 4 Department of Epigenetics and Molecular Carcinogenesis, The University of Texas MD Anderson Cancer Center, Houston, Texas, USA.; 5 Department of Medicine, The University of Texas Health San Antonio, San Antonio, Texas, USA.

**Keywords:** Gastroenterology, Genetics, Oncology, Cancer, Therapeutics, Tumor suppressors

## Abstract

Pancreatic ductal adenocarcinoma (PDAC) occurs as a complex, multifaceted event driven by the interplay of tumor-permissive genetic mutations, the nature of the cellular origin, and microenvironmental stress. In this study, using primary human pancreatic acinar 3D organoids, we performed a CRISPR-KO screen targeting 199 potential tumor suppressors curated from clinical PDAC samples. Our data revealed significant enrichment of a list of candidate genes, with neurofibromatosis type 2 associated gene (*NF2*) emerging as the top target. Functional validation confirmed that loss of *NF2* promoted the transition of PDAC to an invasive state, potentially through extracellular matrix modulation. *NF2* inactivation was found to enhance PDAC cell fitness under nutrient starvation. This adaptation not only reinforced the oncogenic state but also conferred therapeutic resistance. Additionally, we found that *NF2* loss was associated with fibroblast heterogeneity and cancer-stroma communication in tumor evolution. These findings establish *NF2* as a critical tumor suppressor in PDAC and uncover its role in mediating nutrient adaptation and drug resistance. Importantly, this study provides additional insights into drug resistance mechanisms and potential therapeutic targets in PDAC.

## Abstract 2

This study reveals a previously underappreciated gene mutation which could cooperate with environmental context to orchestrate pancreatic cancer development and drug resistance.

## Introduction

Pancreatic ductal adenocarcinoma (PDAC) remains one of the most lethal malignancies, with a dismal 5-year survival rate of approximately 13% (1). Many contributing factors including genetic mutations, the nature of cell of origin, and tumor microenvironmental signaling drive PDAC tumorigenesis (2–4). Over 90% of patients with PDAC harbor oncogenic mutations in *KRAS*, with additional inactivating mutations in tumor suppressor genes including *CDKN2A/p16*, *TP53*, or *SMAD4* commonly observed in patients with PDAC. These oncogenic alterations provide a selective growth advantage that facilitates tumor initiation and progression and, as such, are classified as PDAC driver mutations. Despite recent advances in targeted therapies aimed at oncogenic *RAS* signaling (5, 6), therapeutic strategies targeting other key driver mutations remain limited.

Many patients with PDAC do not carry all 4 key driver mutations simultaneously, suggesting the presence of additional oncogenic events contributing to disease progression. Indeed, next-generation sequencing (NGS) has identified thousands of somatic mutations in PDAC, with over 100 mutated genes on average identified in each patient (7–13). While most of these mutations may represent passenger mutations that do not contribute to PDAC pathogenesis, it is very likely that some previously underappreciated recurrent mutations in patients with PDAC also function as cancer driver mutations. Identifying these potential additional PDAC driver genes is a key step toward understanding tumor biology and developing targeted therapies. In addition, establishing a system that is efficient and cost effective for identification of the causal relationship between gene mutations and therapeutic resistance will offer potential targets to improve treatment outcomes for patients with PDAC.

We have previously reported a flow cytometry–based method to isolate primary acinar and ductal cells from healthy human pancreatic tissues, which can be engineered to recapitulate early development of human PDAC (14–17). Using engineered human cells as a cancer model has inherent advantages over mouse models and established cancer cell lines in the investigation of disease initiation and early progression, thus providing an effective platform to identify additional PDAC drivers in a lineage-specific manner. In the present study, we combined our early human PDAC model with CRISPR screening to perform a KO screen targeting 199 potential tumor suppressor driver mutations in engineered human primary pancreatic acinar cells. Analysis of the screen results identified neurofibromatosis type 2 associated gene (*NF2*) loss as a prominent driver mutation that promotes acinar-derived PDAC progression. Further validation and mechanistic studies revealed that *NF2* inactivation induces intrinsic transcriptomic changes that can prime acinar cells to enhance their fitness under nutrient deprivation, ultimately leading to multidrug resistance.

## Results

### Unveiling of potential cancer driver mutations in tumor suppressors using primary human pancreatic acinar cells.

**Figure 1:**
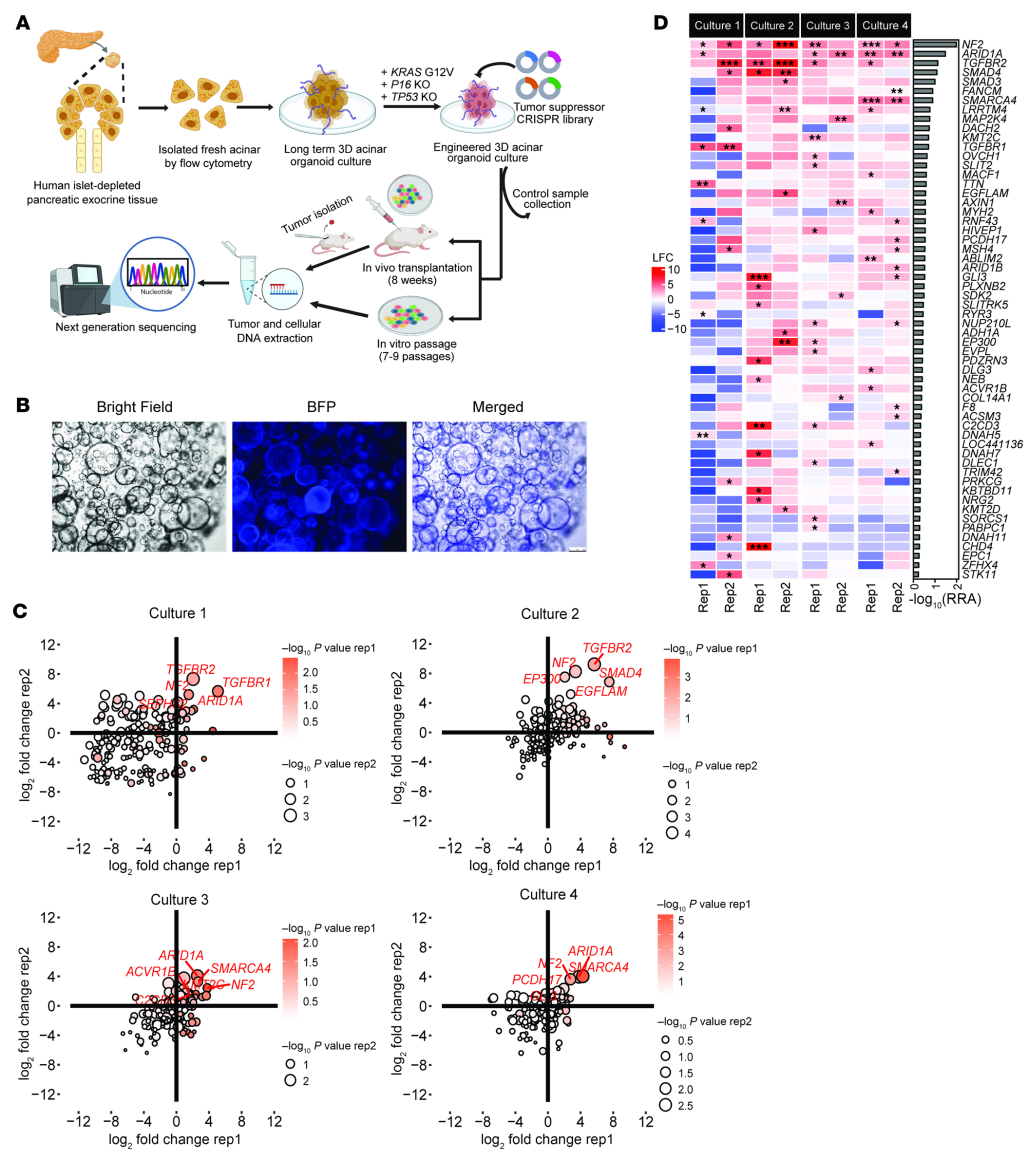
Unveiling of potential tumor suppressor driver mutations in human PDAC using primary pancreatic acinar cells. (**A**) Schematic illustration of CRISPR-KO screen using human primary acinar 3D organoid culture. (**B**) Representative bright-field and fluorescence images of acinar organoids from 4 independent cultures transduced with lentivirus expressing the BFP-tagged CRISPR sgRNA library. Scale bar: 250 μm. (**C**) Scatter plots of the enrichment of target genes from an in vivo CRISPR screen of 4 independent cultures with 2 replicates each. The *x* and *y* axes represent a log_2_ fold change (LFC) of the sgRNA distribution of the indicated target gene in an individual replicate compared with the control. (**D**) Heatmap of all positively enriched target genes in any of the 4 independent cultures. The color scale represents a LFC of the sgRNA distribution of the indicated target gene in an individual replicate compared with the control. The side bar plot indicates the averaged –log_10_ (robust rank aggregation [RRA]) value of each target gene across all replicates. Statistical analyses of the CRISPR screen enrichment were performed using the MAGeCK algorithm.

We used our previously established protocol to isolate primary acinar cells from healthy human pancreatic tissues (14, 15) (see donor information in Supplemental Table 1; supplemental material available online with this article; https://doi.org/10.1172/JCI194395DS1), which were maintained as 3D organoids for long-term culture (Figure 1A). To accelerate acinar transformation for the proposed screen, the organoids were engineered to carry 3 key PDAC driver mutations: overexpression of oncogenic *KRAS^G12V^* and KO of *TP53* and *CDKN2A/p16* (designated as KPT organoids, Supplemental Figure 1, A–C). This combination was determined to be the threshold mutation burden required for acinar transformation in our model (15). Through a comprehensive literature search, we curated a list of 199 potential tumor suppressors recurrently inactivated in clinical PDAC samples (7–13). A pooled CRISPR-KO sgRNA library targeting these genes was constructed, which included a total of 796 sgRNAs with 4 sgRNAs for each target gene (Supplemental Table 2), and the library was validated by NGS for the distribution of all sgRNAs (Supplemental Figure 1D). The library was then packaged into lentivirus and introduced into 4 independently established KPT organoid cultures with duplicates for each (Figure 1B). Following antibiotic selection and a brief expansion, the organoids were transplanted into NSG immunodeficient mice (NOD.Cg-*Prkdc^scid^ IL2Rg^tm1Wjl^*/SzJ, The Jackson Laboratory, strain 005557) for 8 weeks. Tumors were subsequently collected and subjected to NGS.

Analysis of NGS data revealed significant enrichment of sgRNAs targeting 58 genes in at least 1 replicate, suggesting potential advantages in tumor progression upon loss of these target genes (Figure 1, C and D, and Supplemental Table 3). Notably, *SMAD4*, an established PDAC driver that was included in the library as a positive control, was found to be one of the top enriched targets. On the other hand, another positive control target, *TP53*, was not enriched in any of the replicates, as *TP53* inactivation was already introduced in the KPT organoids as our starting materials. These observations together demonstrated the robustness of our methodology. Furthermore, 24 of the 58 genes from the list could be functionally assigned to one of the genetically altered core signaling pathways identified by whole-genome sequencing in patients with pancreatic cancer (7), suggesting their contributions in PDAC development (Supplemental Table 4). In comparison, the implications of the other 34 genes in PDAC are less understood.

Specifically, among all the enriched targets, *NF2*, *ARID1A*, and *TGFBR2* emerged as the top 3 enriched targets that were identified in more than half of the replicates, highlighting their substantial effect on tumor development. *ARID1A* has been previously reported to maintain acinar homeostasis and prevent PDAC transformation in engineered mouse models (18), while loss of *TGFBR2* contributes to tumor development mainly through the TGF-β signaling pathway. In comparison, although the tumor-suppressive role of *NF2* has been established in mesothelioma, schwannomas, and meningiomas, where it is frequently mutated (19, 20), the contributions of *NF2* inactivation to PDAC development remain elusive.

In addition, we also performed in vitro screening to assess the influence of the environmental context on screen outcomes. Transduced organoids were maintained in vitro for 8 weeks (~7–9 passages), followed by DNA extraction and NGS analysis to compare their library distributions with those of freshly transduced cells. This analysis revealed significant enrichment of sgRNAs targeting 31 genes in at least 1 replicate (Supplemental Figure 2, A and B, and Supplemental Table 3). As anticipated, there was a considerable disparity between the enriched target genes identified from the in vitro versus in vivo screen (Supplemental Figure 2C), suggesting a potential interplay between environmental context and genetic predisposition during tumor development. Nevertheless, a total of 13 genes were identified in both settings, with *NF2* remaining as the top hit in both conditions, indicating a strong growth advantage conferred by *NF2* loss in transformed acinar cells.

### Loss of NF2 facilitates aggressive progression of acinar-derived human PDAC.

**Figure 2:**
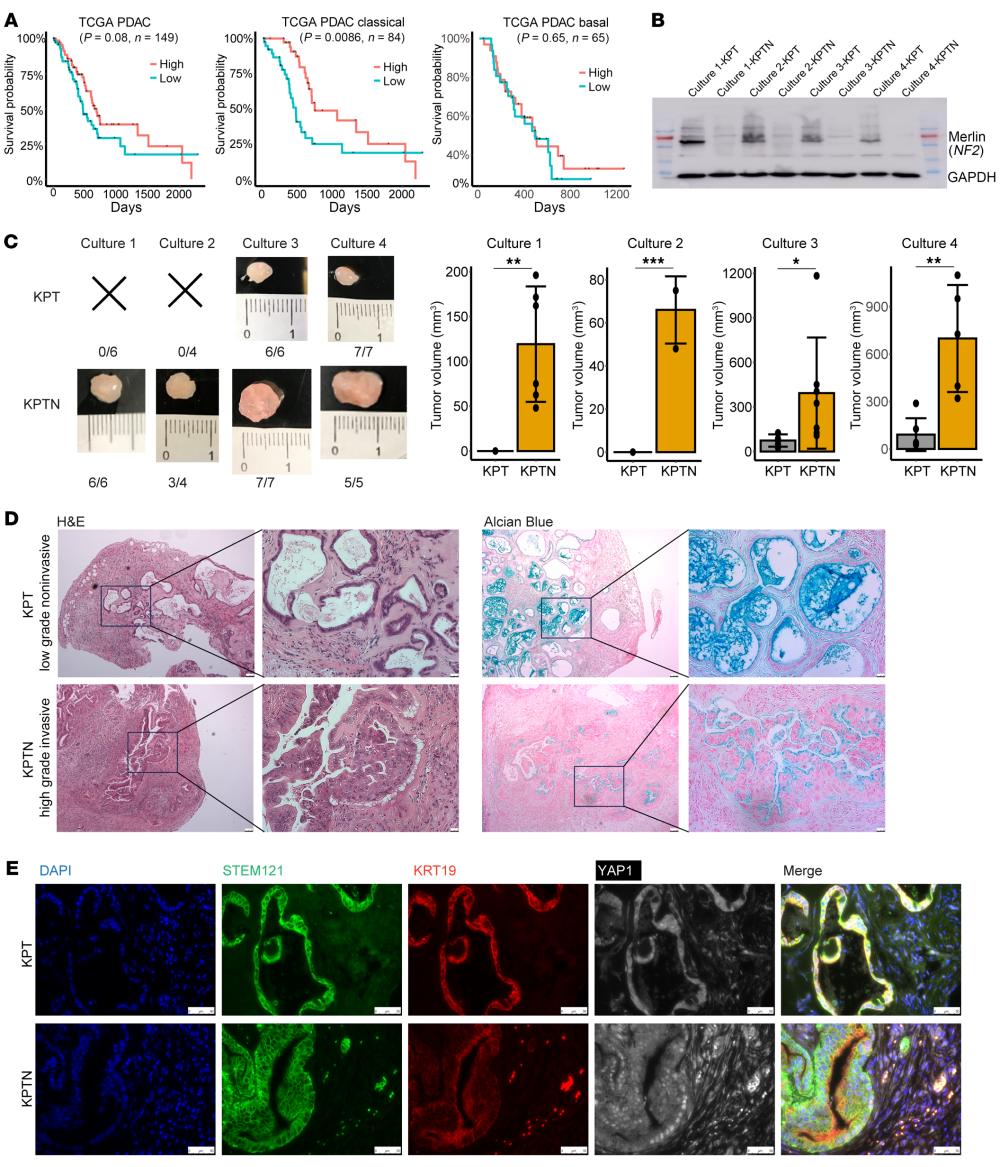
Loss of *NF2* facilitates aggressive progression of acinar-derived human PDAC. (**A**) Kaplan-Meier plots of TCGA PDAC patients (all 149 patients, 84 classical PDAC patients, 65 basal PDAC patients, respectively) separated by median *NF2* expression level. (**B**) Western blot of *NF2* expression in KPT and KPTN organoids derived from 4 independent cultures. (**C**) Representative images of xenograft tumors harvested from NSG mice transplanted with KPT or KPTN organoids derived from 4 independent cultures. The numbers of tumor formed and the total number of transplantations are indicated. Quantification of tumor size. Data represent the SD. *P < 0.05, **P < 0.01, and ***P < 0.001, between 2 genotypes, by 2-tailed Student’s *t* test. (**D**) Representative H&E-stained and Alcian blue staining of tumor tissues as shown in **C** (*n* = 13 KPT tumors, *n* = 21 KPTN tumors). Scale bars: 100 μm and 25 μm (inset). (**E**) Representative immunofluorescence staining of the indicated proteins from tumor tissues as shown in **C** (*n* = 13 KPT tumors, *n* = 21 KPTN tumors). Cell nuclei were counterstained with DAPI. Scale bars: 50 μm.

*NF2* encodes merlin, a cytoskeletal protein that regulates cell-cell contact inhibition and cellular interactions with extracellular matrix (ECM) signaling. Analysis of The Cancer Genome Atlas (TCGA) PDAC cohort revealed that lower *NF2* expression correlates with a poorer prognosis, especially in patients with a classical subtype (21), suggesting a tumor-suppressive role for this gene (Figure 2A). To investigate the contribution of *NF2* inactivation to the development of acinar-derived PDAC, we generated *NF2*-KO acinar cultures (designated as KPTN) in 4 independently established KPT organoid cultures, which were verified by Western blotting at the protein level (Figure 2B). When the KPTN cells were cocultured with KPT cells at a 1:1 ratio in vitro, KPTN cells gradually outcompeted KPT cells and reached dominance by passage 7 (Supplemental Figure 3A). This observation aligns with the growth advantage conferred by *NF2* KO observed in the in vitro CRISPR screen.

To assess the effect of *NF2* inactivation on tumorigenicity, the engineered organoids were subcutaneously transplanted into NSG mice. KPT cells derived from cultures 1 (*n* = 6) and 2 (*n* = 4) failed to form tumors within 8 weeks of inoculation. In contrast, *NF2* KO in these cultures resulted in successful tumor formation (*n* = 6 and 4, respectively) (Figure 2C and Supplemental Figure 3B). Notably, while xenograft tumors were successfully established by transplanting KPT cells from cultures 3 (*n* = 6) and 4 (*n* = 7), *NF2* inactivation significantly increased the tumor size compared with their KPT counterparts (*n* = 7 and 5, respectively). Histological analysis of tumor tissue sections revealed that KPTN tumors progressed to high-grade lesions characterized by nuclear atypia, whereas all KPT tumors remained low-grade lesions with substantial Alcian blue staining (Figure 2D). The less aggressive phenotype from our KPT transplantation is in line with a previous report using a similar engineered pancreatic organoid model (22). Immunofluorescence staining with a human-specific STEM121 antibody marked the tumor cells of human origin (Figure 2E and Supplemental Figure 3C). The presence of scattered STEM121^+^ human cells at sites spatially separated from the primary tumor lesion provides additional evidence for invasive cancer cell populations in KPTN tumors (Figure 2E and Supplemental Figure 3C). These findings together suggested that loss of *NF2* facilitated tumor development and promoted malignant progression of acinar-derived PDAC. Consistent with our previous findings (15), all the acinar-derived tumor cells were associated with strong expression of KRT19, a marker of normal ductal lineage not expressed in healthy acinar cells. In addition, in line with the established role of *NF2* in the hippo pathway, KPTN tumors showed abundant nuclear YAP1 staining (Supplemental Figure 3D), further validating the functional consequences of *NF2*-KO.

### Molecular dissection of the NF2 loss–induced tumor evolution in the in vivo context.

**Figure 3:**
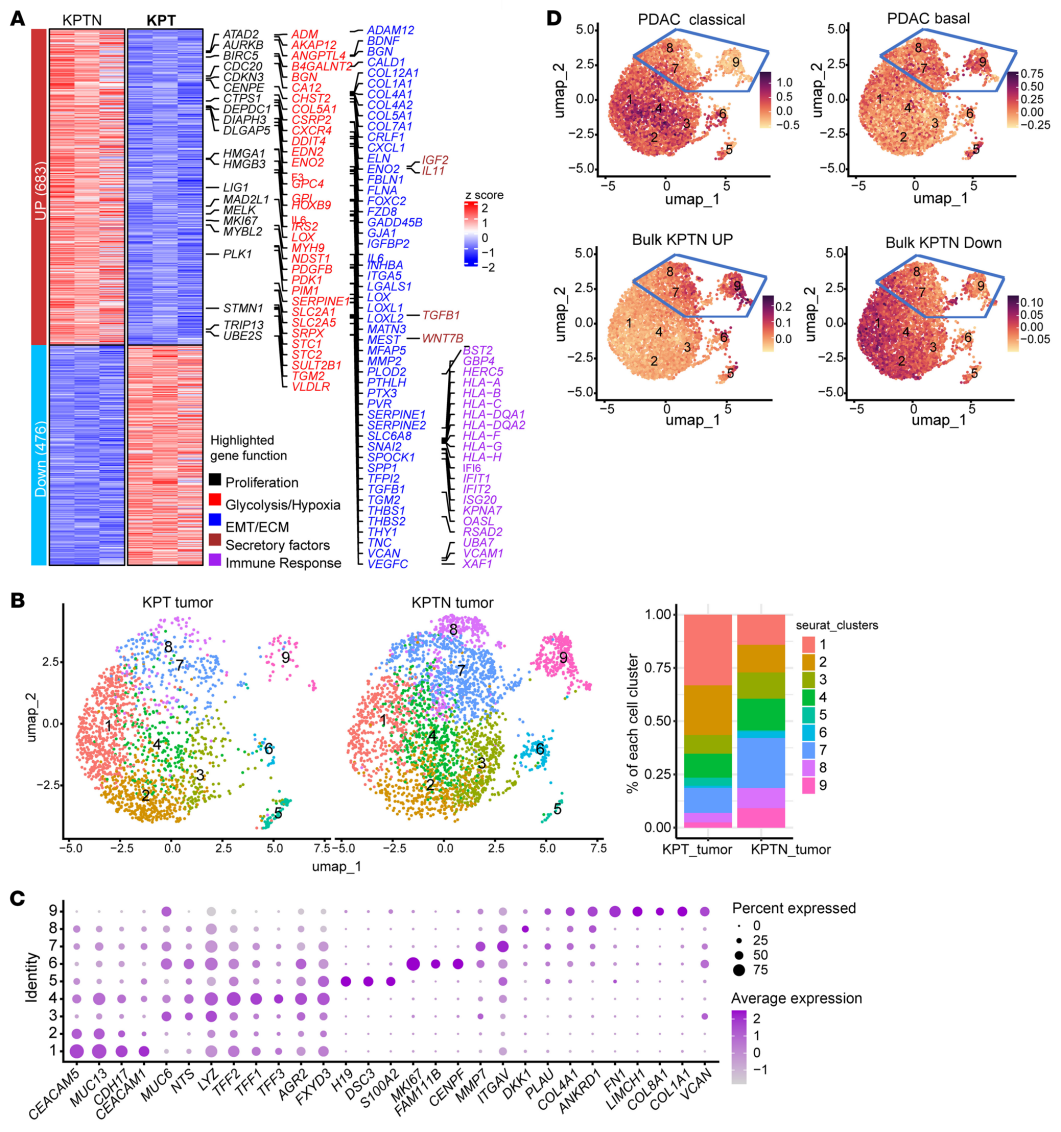
Molecular dissection of *NF2* loss–induced tumor evolution in the in vivo context. (**A**) Expression heatmap of DEGs in KPTN versus KPT tumors (*n* = 3 in each group (fold change >2, *P_adj_* < 0.05). Side annotation includes genes involved in specific functional pathways as indicated at the bottom. (**B**) Left: Uniform manifold approximation and projection and (UMAP) of cells of human origin present in KPT- and KPTN-derived tumor tissues in the scRNA-Seq analysis. Right: Composition percentage of each cell population. (**C**) Expression of the indicated feature genes in each cell population as shown in **B**. (**D**) Expression level of the indicated gene modules in all the populations shown in **B**. The bulk KPTN upregulated (Up) and bulk KPTN downregulated (Down) genes were curated from the analysis shown in **A**.

To understand how *NF2* inactivation can promote acinar-derived PDAC transformation, bulk RNA-Seq was performed to detect global transcriptional alterations in KPTN versus KPT-derived tumor tissues (*n* = 3). We identified 683 human genes significantly upregulated in KPTN tumors (fold change >2, adjusted *P* [*P_adj_*] < 0.05), which are involved in proliferation, glycolysis, hypoxia, focal adhesion, as well as collagen formation, suggesting a more active cancer progression (Figure 3A, Supplemental Figure 4A, and Supplemental Table 5). Conversely, 476 genes downregulated in KPTN tumors included immune-related genes, consistent with our previous report (15), highlighting a possible intrinsic immune evasion mechanism during PDAC progression (Supplemental Figure 4A).

Single-cell RNA-Seq (scRNA-Seq) analysis was further performed to assess the heterogeneity in KPT- and KPTN-derived tumor tissues (Supplemental Figure 4, B and C). Clustering of 4,985 human cells revealed 9 shared populations, with clusters 7–9 predominantly present in KPTN tumors (Figure 3B and Supplemental Table 6). These clusters overexpressed genes involved in ECM remodeling (*MMP7*, *ITGAV*, *COL4A1*, *COL1A1*, *FN1*, *VCAN*, etc.) and YAP signaling (*ANKRD1*) (Figure 3C). Expression levels of these genes were inversely correlated with a classical PDAC gene signature and positively associated with a basal subtype gene signature (Figure 3D), linking *NF2* loss to a relatively more aggressive phenotype in acinar-derived PDAC. Notably, although our KPTN tumors were associated with a higher basal subtype gene signature relative to KPT tumors, when compared with TCGA PDAC samples, they still exhibited a phenotype more closely resembling the clinical classical subtype (Supplemental Figure 5A). Given that lower *NF2* expression is associated with a worse prognosis in classical PDACs (Figure 2A), our observations suggest that *NF2* loss may promote disease progression during the less aggressive PDAC stage recapitulated in our experimental model. Additionally, projection of bulk RNA-Seq KPTN signatures onto scRNA-Seq data linked *NF2* loss–induced transcriptomic changes to clusters 7–9 (Figure 3D), whose gene signatures are highly expressed in more aggressive TCGA PDAC samples (basal and high-grade) (Supplemental Figure 5B and Supplemental Table 7).

**Figure 4:**
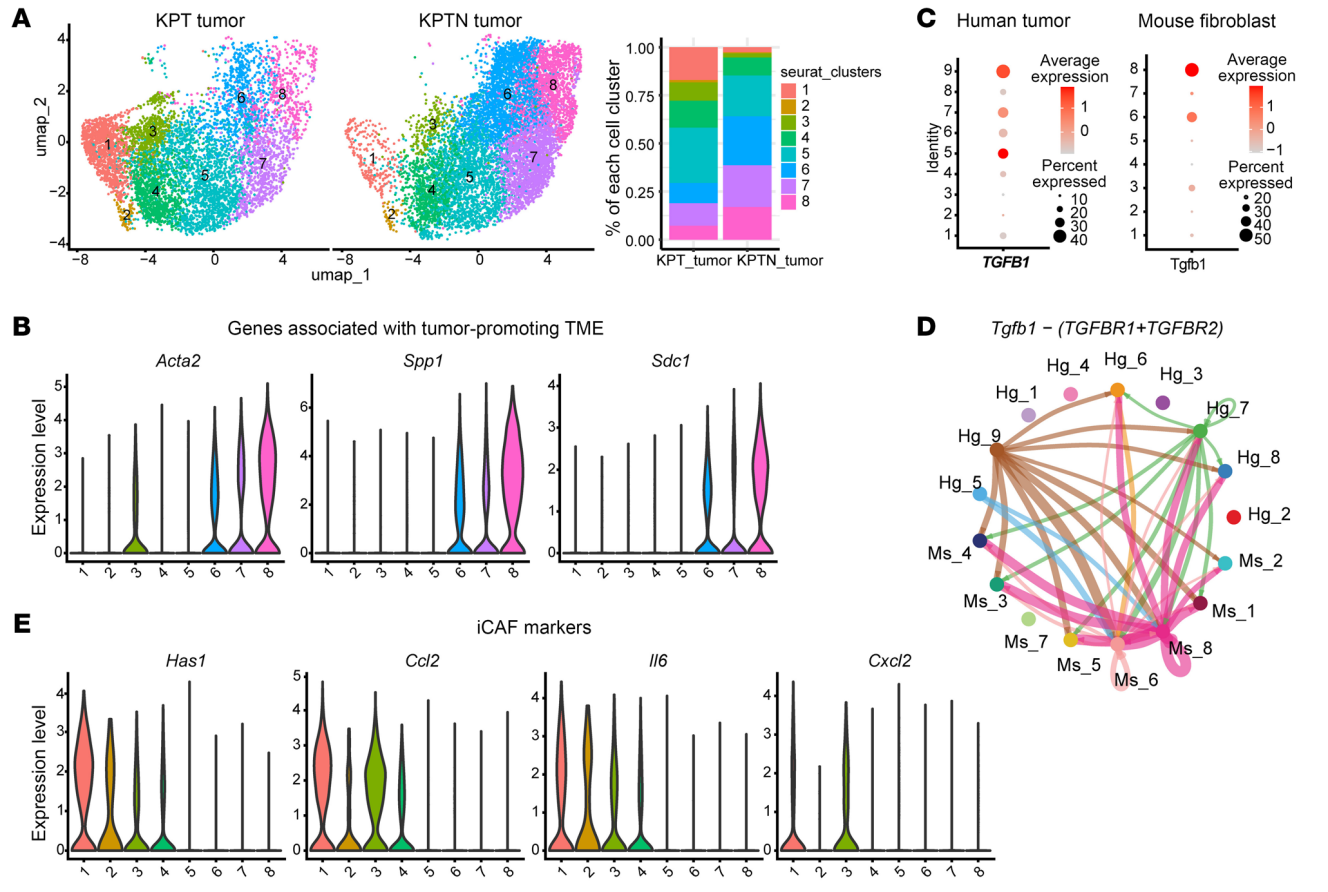
KPTN tumors are associated with a tumor-promoting microenvironment. (**A**) UMAP of all mouse fibroblasts present in KPT- and KPTN-derived tumor tissues and the composition percentage of each cell population. (**B**) Violin plots showing expression of the indicated genes associated with a tumor-promoting TME in each cell population as shown in **A**. (**C**) *TGFB1* and *Tgfb1* gene expression in each cell population of human and mouse origin shown in Figure 3B and Figure 4A. (**D**) Inferred cell-cell communication via *TGFB1* signaling among cell populations shown in Figure 3B and Figure 4A. (**E**) Violin plots showing expression of the indicated genes associated with iCAFs in each cell population as shown in **A**.

Analysis of mouse cancer-associated fibroblasts (CAFs) from tumor tissues revealed a total of 8 clusters of cells (Figure 4A and Supplemental Table 6). Interestingly, fibroblasts from KPTN tumors were enriched with populations (clusters 6–8) that highly expressed *Acta2*, *Spp1*, and *Sdc1*, which were associated with a tumor-promoting fibroblast phenotype (Figure 4B) (23–25). It has been established that TGF-β signaling in the PDAC tumor microenvironment (TME) contributes to the activation of myofibroblast-like CAFs (myCAFs) to foster a more aggressive PDAC phenotype (26–28). Here, we observed higher *TGFB1* and *Tgfb1* expression in human tumor cluster 9 and mouse fibroblast cluster 8, respectively, which were both enriched populations in KPTN samples (Figure 4C). Consistently, cell-cell communication analysis also inferred an enhanced TGF-β signaling from these 2 clusters (Figure 4D), which probably contributes to a tumor-promoting microenvironment in KPTN tumors. In comparison, mouse fibroblasts from KPT tumors were enriched with cells highly expressing tumor-suppressing inflammatory CAF (iCAF) markers, including *Has1*, *Ccl2*, *Il6*, and *Cxcl2* (clusters 1–3) (Figure 4E and Supplemental Figure 5C) (29). Additionally, cells in mouse clusters 1–3 also had high expression of a recently reported complement-secreting CAF (csCAF) gene signature (Supplemental Figure 5C and Supplemental Table 7) (30).

### Genetic predisposition primes acinar cells for enhanced cell fitness in the tumor environment.

**Figure 5:**
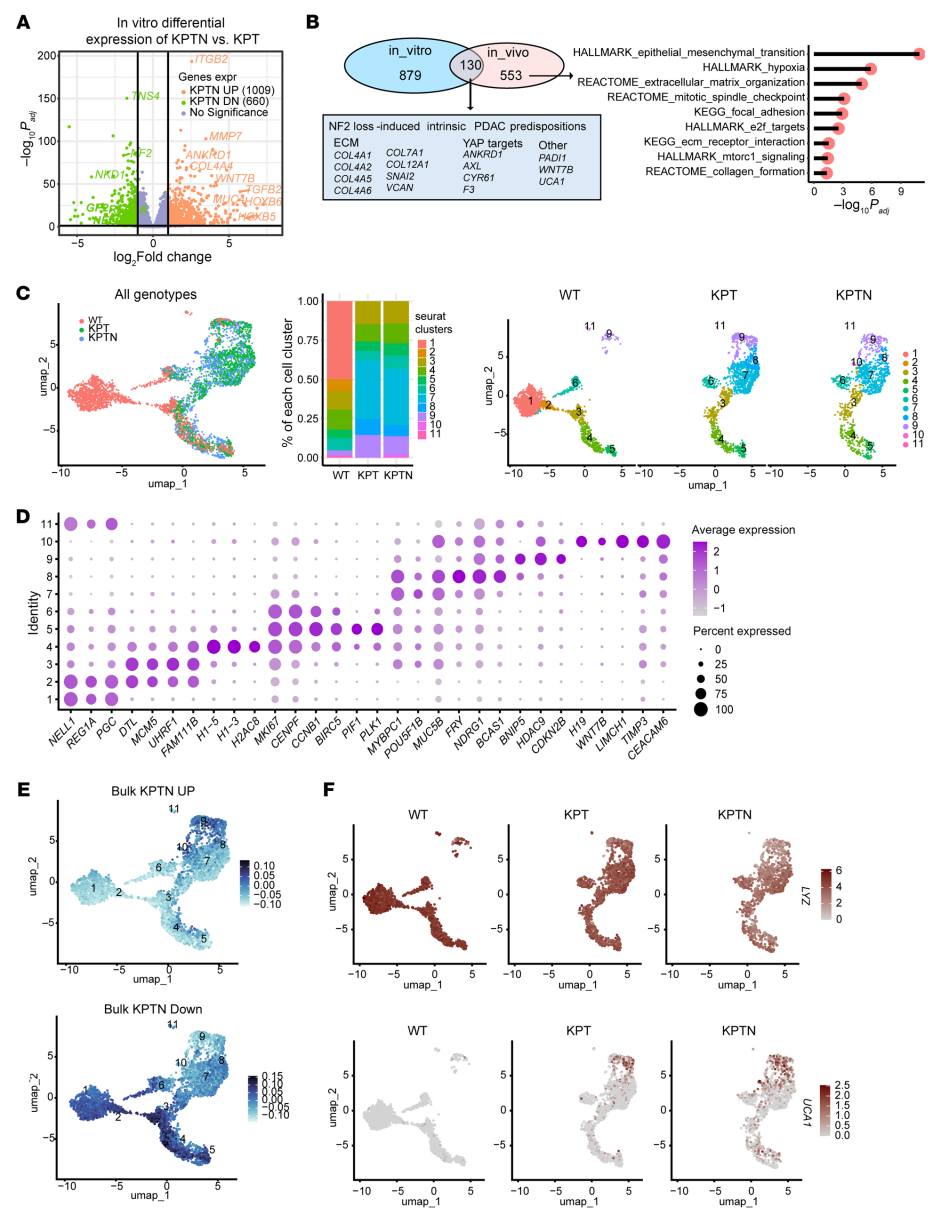
Genetic predisposition primes acinar cells for enhanced cell fitness in the tumor environment. (**A**) Volcano plot of DEGs in KPTN versus KPT in vitro organoids (*n* = 6 for each genotype). (**B**) Left: Overlap of the upregulated genes in KPTN in vitro cultures and KPTN xenograft tumors (as compared with their KPT analogs, respectively). Left: Boxed blue area indicates representative genes among the 130 overlapping genes. Right: Overrepresentation analysis of the 553 genes that were only upregulated in KPTN tumors, but not in the in vitro culture. (**C**) Left: UMAP of all cells from WT, KPT, and KPTN in vitro cultures in the scRNA-Seq analysis. Middle: Percentage composition of each cell population in samples from each genotype. Right: UMAP of cells split by each individual genotype. (**D**) Expression of the indicated feature genes in each cell population as shown in **C**. (**E**) Expression levels of the indicated gene modules in the cell populations shown in **C**. The bulk KPTN upregulated and bulk KPTN downregulated genes were curated from the analysis shown in **A**. (**F**) Expression levels of the indicated genes in the cell populations shown in **C**.

Although organoids are widely used in cancer studies (31), their ability to mimic the in vivo TME remains unclear. To address this, we performed RNA-Seq analysis on KPT and KPTN organoids, which revealed 1,009 upregulated and 660 downregulated genes in KPTN cells compared with their KPT counterparts (fold change >2, *P_adj_* < 0.05) (Figure 5A and Supplemental Table 5). A comparison of these with the 683 KPTN tumor–upregulated genes (as described in Figure 3A) revealed 130 consistently upregulated genes (Figure 5B), including ECM components (*COL4A1*, *COL4A2*, *COL12A1*, *VCAN*, etc.) and YAP targets (*ANKRD1*, *AXL*, *CYR61*, *F3*) (32). In comparison, other upregulated genes in KPTN samples were found to be context specific. The 553 genes specifically upregulated in tumors were enriched in hypoxia, glycolysis, EMT, focal adhesion and proliferation pathways, reflecting a state shift to adapt to the hostile in vivo environment. Together, these findings suggested that *NF2* inactivation may trigger intrinsic transcriptomic alterations that prime acinar cells for enhanced fitness in the TME.

To further probe the heterogeneity in the in vitro cultures, we performed scRNA-Seq on WT, KPT and KPTN organoids and found 11 cell clusters (Figure 5C and Supplemental Table 6). While all 3 genotypes share proliferating cell clusters (clusters 3–6) featuring high expression of cell-cycle and DNA replication genes, WT organoids have unique cell populations including 2 major *REG1A*^+^*PGC*^+^ cluster 1 and 2 cells, representing an acinar-to-metaplastic cell-transitioning stage (Figure 5D). In comparison, the unique populations in engineered cells (clusters 7–10) lost all acinar markers and were associated with gene expression found in cancer cells including *MUC5B* and *CEACAM6* (33, 34). Comparison with bulk RNA-Seq data (as shown in Figure 5A) demonstrated that cells in clusters 1–6 highly expressed KPTN downregulated genes, while cells in clusters 8–10 highly expressed KPTN upregulated genes (Figure 5E). Pseudotime trajectory analysis predicted that the WT metaplastic acinar cells bifurcate toward a proliferating or cancer-like state (Supplemental Figure 6A). The acinar cells gradually lost the classical PDAC subtype gene signature (e.g., *LYZ*, *TFF2*, and *AGR2*) upon acquiring driver mutations in the in vitro context, whereas high expression of *UCA1*, a basal PDAC subtype marker, was observed in engineered cells (Figure 5F and Supplemental Figure 6B).

**Figure 6:**
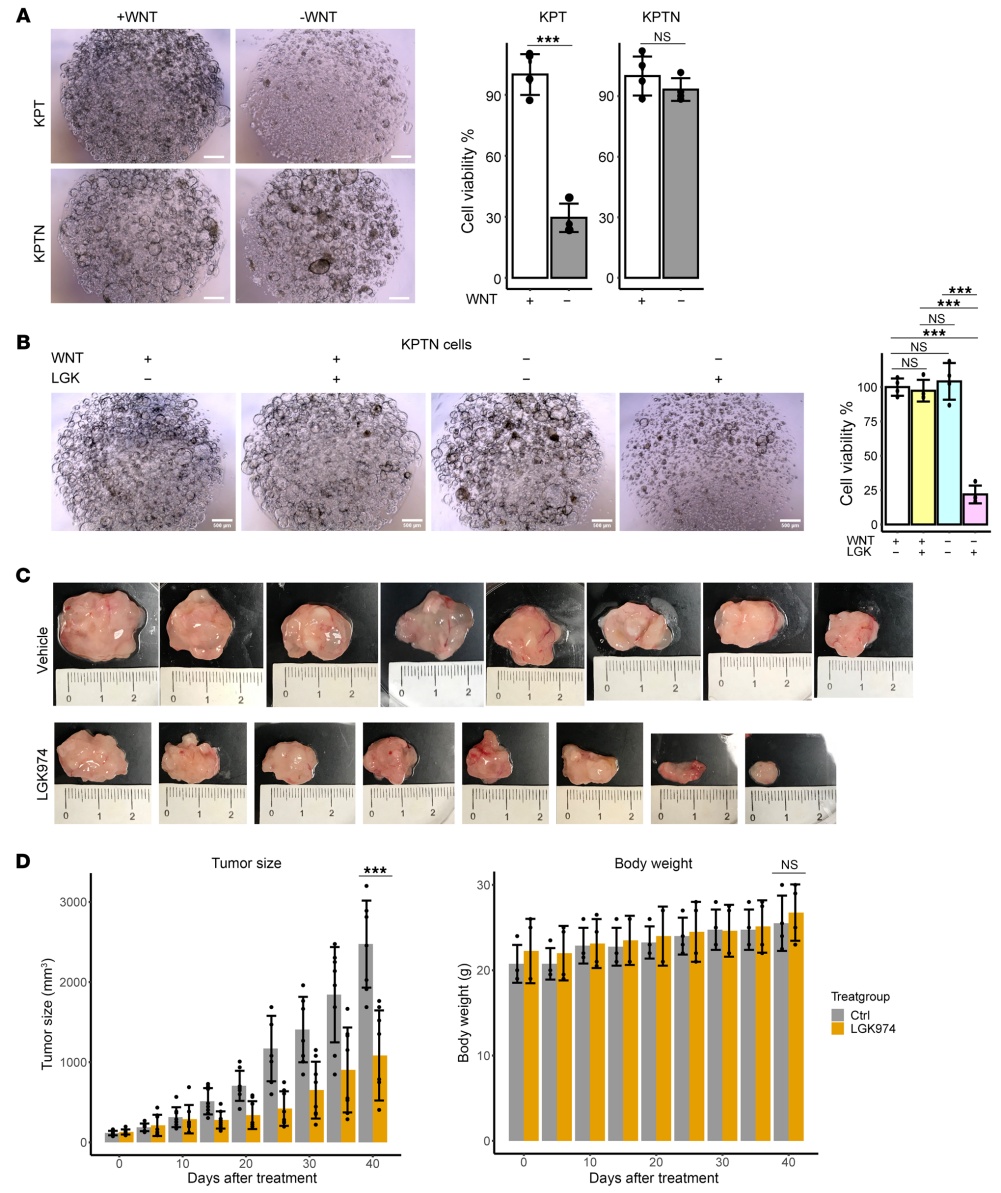
*NF2* loss drives autonomous WNT7B secretion to support acinar growth. (**A**) Left: Representative images of KPT and KPTN in vitro cultures incubated with or without WNT3A supplement from 4 independent biological replicates. Scale bars: 500 μm. Right: Quantification of cell viability. ***P < 0.001, between the 2 groups, by 2-tailed Student’s *t* test. Error bar represents the SD. (**B**) Left: Representative images of KPTN in vitro culture incubated with or without WNT3A supplement or LGK974 (100 nM) from 4 independent biological replicates. Scale bars: 500 μm. Right: Quantification of cell viability. ***P < 0.001, by 1-way ANOVA followed by multiple pairwise comparison using Tukey’s honest significant Differences test. Data represent the SD. (**C**) Photographs of xenograft tumors from NSG mice transplanted with KPTN tumors and given a daily dose of LGK974 (10 mg/kg, oral gavage) or vehicle control. (**D**) Tumor size and body weight comparisons between mice that received LGK974 or vehicle control. ***P < 0.001, between the 2 groups at the indicated time point, by 2-tailed Student’s *t* test. Error bar represents the SD. .

Interestingly, bulk RNA-Seq data revealed *WNT7B* upregulation in both KPTN tumors and organoids, where it functions as an essential niche factor for maintaining human pancreatic organoid cultures and supporting tumor growth (22, 35). To functionally verify the implication of *WNT7B* upregulation, we cultured organoids in WNT3A-free medium and observed reduced growth from KPT organoids, whereas KPTN organoids remained unaffected (Figure 6A). Additionally, treatment with 100 nM LGK974, a WNT protein secretion inhibitor, dramatically suppressed KPTN organoid growth in WNT3A-free medium but not in WNT3A-supplemented medium (Figure 6B). This suggested that *NF2* loss drives autonomous WNT7B secretion, resulting in enhanced cell survival in WNT-deprived conditions. Although *GATA6* dysregulation has been linked to elevated WNT ligand expression in PDAC (22), its expression was similar between KPT and KPTN samples (Supplemental Figure 6C), suggesting the existence of alternative mechanisms in the regulation of *WNT* expression. These findings establish that *NF2* inactivation enables tumor cell survival independent of exogenous WNT signals and promotes tumor progression.

To test the therapeutic potential of WNT inhibition, we treated KPTN xenografts in NSG mice with LGK974 (10 mg/kg daily) (36). After 40 days, LGK974 significantly reduced tumor size compared with the vehicle treatment group, without significant changes in mouse body weight (Figure 6, C and D). In addition, the xenograft tumor cells remained sensitive to LGK974 treatment (100 nM) after being harvested and recultured in vitro (Supplemental Figure 6D), suggesting that minimal resistance developed during the in vivo tumor progression.

### NF2 inactivation promotes cell survival under nutrient deprivation via enhanced macropinocytosis.

**Figure 7:**
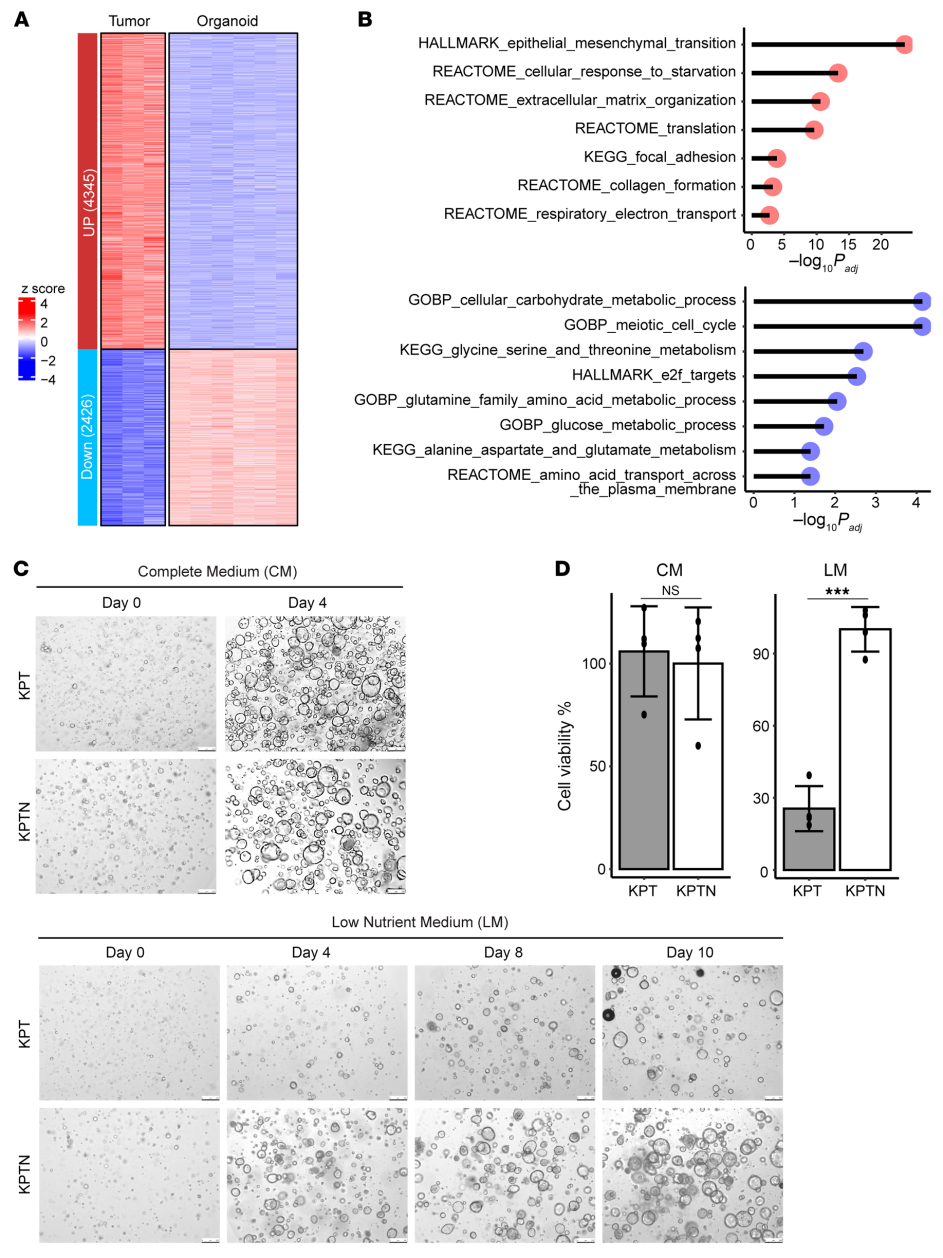
*NF2* inactivation promotes cell survival under nutrient deprivation. (**A**) Expression heatmap of DEGs in KPTN tumors (*n* = 3) versus KPTN in vitro organoid cultures (*n* = 6). Genes were defined by a fold change >2 and *P_adj_* < 0.05. (**B**) Overrepresentation analysis for upregulated (in red) and downregulated (in blue) genes in KPTN tumors. (**C**) Representative images of KPT and KPTN organoids cultured in CM or LM for the indicated durations from 4 biological replicates. Scale bars: 250 μm. (**D**) Quantification of cell viability of the organoids shown in **C**. ***P < 0.001 between the 2 groups, by 2-tailed Student’s *t* test. Data represent the SD.

To assess the effect of the environment on acinar transformation, we compared bulk RNA-Seq data on KPTN tumors and organoid cultures upregulation of genes involved in the cellular response to starvation and downregulation of genes responsible for glucose and amino acid metabolism in tumor cells (Figure 7, A and B, and Supplemental Table 5). Those transcriptomic changes reflected metabolic rewiring during tumor progression under nutrient-scarce in vivo conditions. Comparison of environmentally induced genes (as shown in Figure 7A) with the genetically induced genes (as shown in Figure 3A) identified a total of 378 genes that seemed to be primed by genetic predisposition and reinforced by environmental stress (Supplemental Figure 7A). This list included many genes involved in ECM remodeling and YAP signaling, as well as secreted proteins associated with cancer development.

Analysis of TCGA PDAC data showed that a higher expression score for the induced starvation response genes in KPTN tumors was associated with a worse prognosis (Supplemental Figure 7B and Supplemental Table 7). Additionally, we observed that *NF2* expression correlated with alterations in multiple cellular metabolism programs in TCGA PDAC patients (Supplemental Figure 7B). On the basis of these findings, we hypothesized that loss of *NF2* contributes to acinar-derived PDAC progression by enhancing cell survival in a nutrient-deprived environment. To test this, we cultured KPT and KPTN organoids in low-nutrient medium (LM) and complete-nutrient medium (CM) (see Methods) and monitored for cell growth. While KPT and KPTN cultures showed comparable viability in CM during short-term culturing (Figure 7, C and D), KPT cells ceased growing in LM after extended culture periods. In contrast, KPTN cells underwent an initial growth arrest but resumed proliferation by days 6–8 in LM.

**Figure 8:**
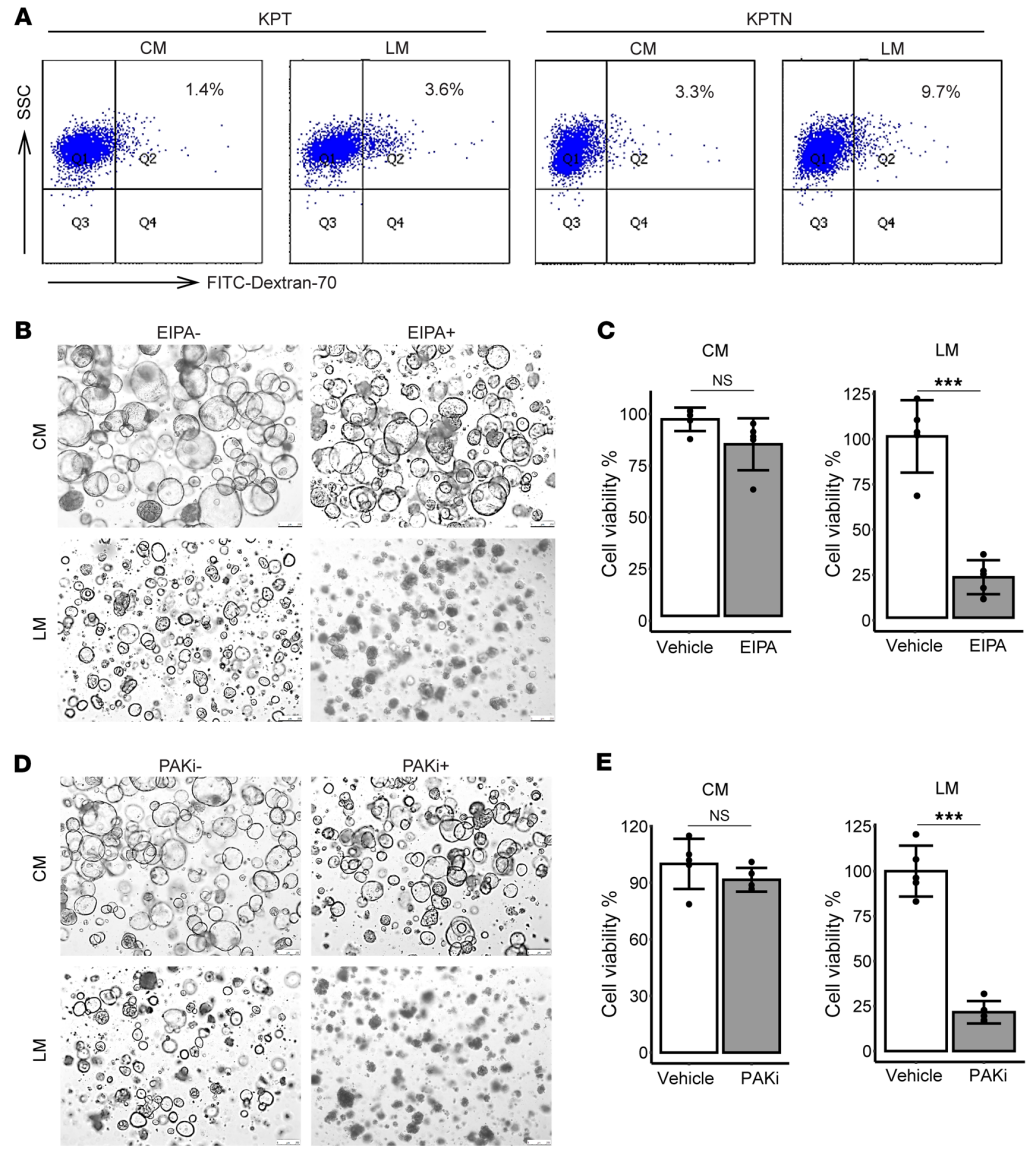
*NF2* loss enhances macropinocytosis activation under starvation. (**A**) Representative set of flow cytometric analyses of FITC–dextran 70 uptake in KPT and KPTN organoids cultured in CM or LM (*n* = 4 biological replicates). (**B**) Representative images of KPTN organoids cultured in CM or LM and treated with or without EIPA (2.5 μM) from at least 4 biological replicates. Scale bars: 250 μm. (**C**) Quantification of cell viability in the organoids shown in **B**. ***P < 0.001, between 2 groups, by 2-tailed Student’s *t* test. Data represent the SD. (**D**) Representative images of KPTN organoids cultured in CM or LM and treated with or without 15 nM PAK inhibitor (*n* = at least 4 biological replicates). Scale bars: 250 μm. (**E**) Quantification of cell viability of the organoids shown in **D**. ***P < 0.001, between 2 groups, by 2-tailed Student’s *t* test. Data represent the SD.

Macropinocytosis represents one of the crucial mechanisms for cancer cells to combat nutrient starvation (37, 38). It was also reported that Merlin regulates growth factor–induced macropinocytosis for membrane receptor recycling (39). Given these facts, we proposed that *NF2* loss could activate macropinosytosis for nutrient scavenging under starvation conditions. To test this, KPT and KPTN organoids were fed FITC-conjugated dextran 70 for 1 hour and then analyzed by flow cytometry. When cultured in CM, KPTN cells exhibited a higher basal level of dextran uptake compared with KPT cells (3.3% vs. 1.4%) (Figure 8A). Importantly, when exposed to LM for 48 hours, dextran uptake was detected in 9.7% of KPTN cells compared with 3.6% of KPT cells, suggesting enhanced activation of macropinocytosis in KPTN cells under nutrient deprivation.

To further examine the contribution of macropinocytosis to cell survival in low-nutrient environments, we cultured the KPTN organoids in CM or LM with or without supplementation of ethylisopropylamiloride (EIPA), a macropinocytosis inhibitor (37, 40). The results showed that EIPA treatment (2.5 μM) dramatically reduced the survival of KPTN cells in LM, while having only a minor effect on the cells in CM (Figure 8, B and C). This observation suggested that, in addition to previously reported growth factor–induced macropinocytosis, loss of *NF2* also primed cells for macropinocytosis activation upon exposure to nutrient deprivation stress. Consistently, we found that overexpression of Merlin in the established PDAC cell line Panc1 suppressed the macropinocytosis induced by nutrient scarcity (Supplemental Figure 7, C and D).

Protein-protein interaction analysis using the STRING database (41) revealed 99 proteins reported or predicted to interact with *NF2* (Supplemental Figure 7E). As expected, a subset of these proteins are involved in hippo pathways. Other identified proteins are mainly involved in a few categories including tumor suppression, cell growth regulation, as well as chromatin remodeling. Additionally, the analysis revealed an interaction between *NF2* and PAK1 (p21-activated kinases). Interestingly, loss of function in *NF2* has been reported to trigger PAK1 activation, which was shown to promote glutamine deprivation–induced macropinocytosis in a PDAC cell line (42, 43). To test whether PAK1 can mediate macropinocytosis activation upon *NF2* loss, KPTN organoids were cultured in CM or LM with or without FRAX597, a PAK inhibitor (44). The results showed that treatment of FRAX597 at 15 nM completely abolished KPTN survival in LM, while only moderately affecting cell fitness in CM (Figure 8, D and E). This finding demonstrated that inhibiting PAK1 attenuated starvation-induced macropinocytosis upon *NF2* loss, highlighting an *NF2* loss/PAK1 activation–enhanced macropinocytosis axis during pancreatic acinar transformation under nutrition scarcity.

### Genetic events and environmental cues orchestrate to induce therapeutic resistance.

**Figure 9:**
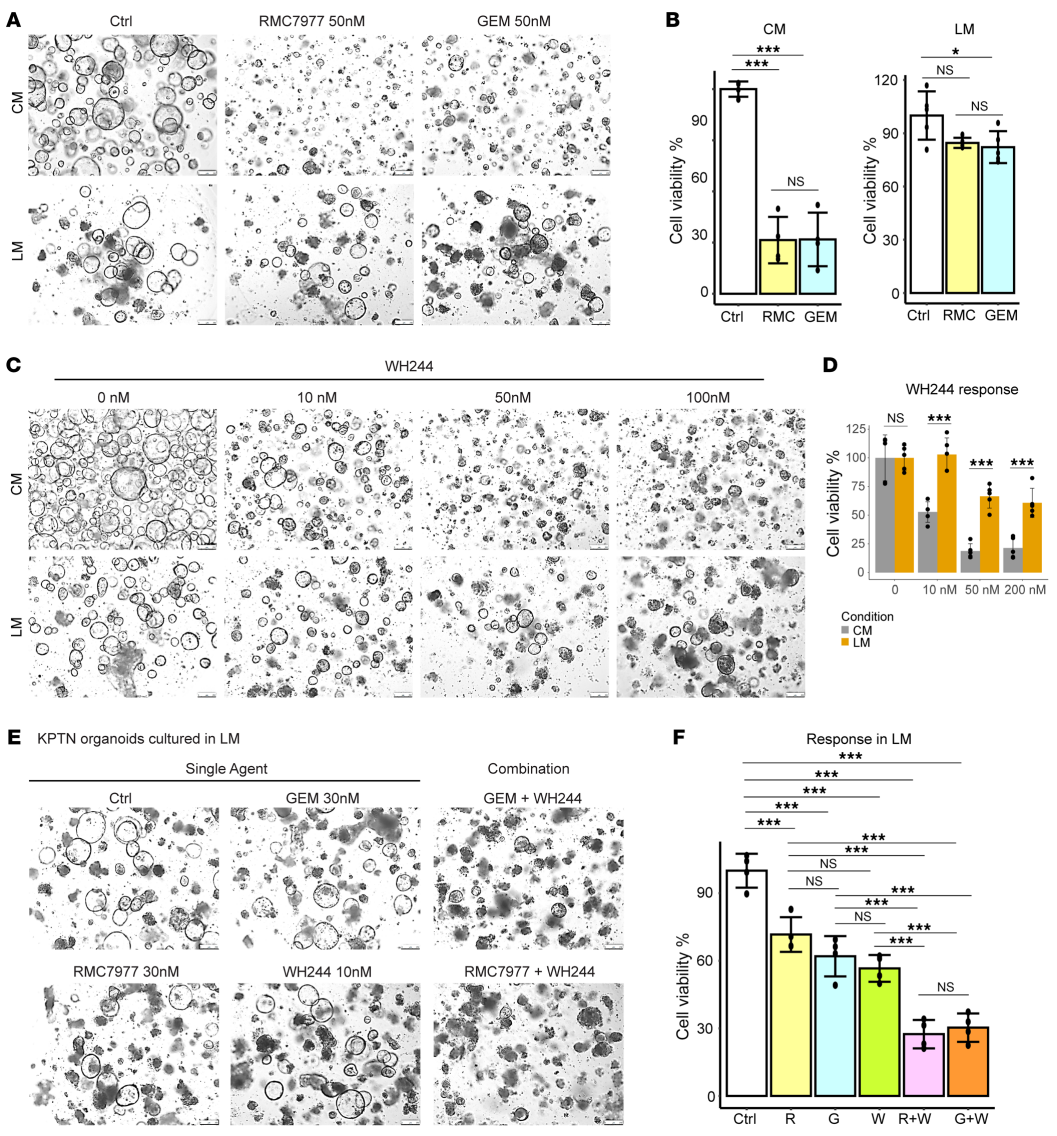
*NF2* loss and nutrient starvation cooperate to induce therapeutic resistance. (**A**) Representative images of KPTN organoids cultured in CM or LM and treated with RMC 7977, gemcitabine, or vehicle control (*n* = at least 4 independent experiments). Scale bars: 250 μm. (**B**) Quantification of the cell viability shown in **A**. *P < 0.05 and ***P < 0.001, by 1-way ANOVA followed by multiple pairwise comparison using Tukey’s honest significant differences test. Error bar represents the SD. (**C**) Representative images of KPTN organoids cultured in CM or LM and treated with WH244 or vehicle control (*n* = at least 4 biological replicates). Scale bars: 250 μm. (**D**) Quantification of cell viability as shown in **C**. ***P < 0.001, between CM versus LM at the same dosage, by 2-tailed Student’s *t* test. Data represent the SD. (**E**) Representative images of KPTN organoids cultured in LM and treated with the indicated single or combination regime or with vehicle control (*n* = 4 biological replicates). Scale bars: 250 μm. (**F**) Quantification of cell viability of the organoids shown in **E**. ***P < 0.001, by 1-way ANOVA followed by multiple pairwise comparison using Tukey’s honest significant differences test. Error bar represents the SD.

Resistance to therapeutic treatment has been a major obstacle for improving PDAC outcomes. Here, we observed that the increased driver mutation burden in engineered acinar cells resulted in enhanced resistance to RMC7977 (at 10 nM to 1 μM), a recently characterized pan-RAS inhibitor (5), highlighting the contribution of a genetic predisposition to drug resistance (Supplemental Figure 8A). Interestingly, it was reported that different in vitro culture environments could alter the cell states and affect drug responses in patient-derived PDAC organoids (45). We reasoned that the KPTN organoids that survive in a nutrient-deprived environment may undergo a phenotypic shift, which could result in further altered drug responses. To test this idea, KPTN cells were allowed to be fully acclimated in LM for 10 days and then treated with gemcitabine and RMC7977 at various doses (Supplemental Figure 8B). The cells continuously cultured in CM were subjected to the same treatment and served as controls. The results showed that, while both gemcitabine and RMC7977 were lethal to KPTN organoids at 50 nM, the LM-acclimated KPTN cells showed only limited responses (Figure 9, A and B, and Supplemental Figure 8, C and D).

Dysregulation of apoptosis has been one of the cancer hallmarks and is linked to therapeutic resistance in cancer (46, 47). To test whether inducing apoptosis can reverse the observed drug resistance, we tested a recently developed Bcl-2/Bcl-xL dual degrader (WH244) (48) in KPTN organoids cultured in LM or CM. Although WH244 alone showed limited efficacy against LM-acclimated KPTN organoids (Figure 9, C and D), the combination of WH244 with gemcitabine or RMC7977 led to an improved inhibitory effect compared with single treatment (Figure 9, E and F). Taken together, our findings revealed that *NF2* inactivation enhanced cell survival and therapy resistance under nutrient deprivation, likely through metabolic rewiring and macropinocytosis activation.

## Discussion

While *KRAS*, *TP53*, *CDKN2A/p16*, and *SMAD4* are the most frequently mutated genes in PDAC, most tumors do not harbor all these mutations, suggesting the existence of other driver mutations. In this study, we used our human model of early-stage PDAC combined with CRISPR screening to identify additional tumor suppressor driver mutations that may offer potential treatment options, and discovered *NF2* inactivation as a potent driver of acinar-derived PDAC. Our results demonstrated that *NF2* loss facilitated the aggressive progression of human pancreatic acinar cell–derived PDAC. Mechanistically, *NF2* inactivation induced intrinsic PDAC-associated transcriptomic changes, including alterations in ECM components and elevated niche factor expression, which predisposed cells for tumor initiation and progression. Additionally, *NF2* loss primed acinar cells for enhanced cell fitness in hostile environments, such as nutrient deprivation and therapeutic stress. Our findings demonstrate the tumor-suppressive role of *NF2*, which is consistent with a previous study showing that *NF2* overexpression in PDAC cell lines suppresses cell growth (49). Importantly, our present study using primary pancreatic acinar organoids has inherent advantages over established cancer lines in interrogating the complex interplay of genetic events, the nature of cellular origin, and the environmental context during PDAC early development.

In the present study, we observed heterogeneity in the CRISPR screen results from each independently established culture. This may be attributed to the inherent genetic variability of the corresponding donor origins. Supporting this explanation, a previous scRNA-Seq study demonstrated that patient-derived PDAC organoid samples clustered according to their individual donor origin (50). Moreover, the differentially expressed genes (DEGs) in each organoid sample were found to be patient specific and included diverse gene families such as those involving cell-surface and transmembrane proteins, secreted proteins, and metabolic enzymes. This observation highlights the idea that heterogeneity in patient-derived organoids occurs at a whole-transcriptome level.

RNA-Seq analysis and validation experiments confirmed that *NF2* inactivation intrinsically upregulated *WNT7B* expression and protein secretion, which can be targeted to suppress early PDAC development. While *GATA6* dysregulation has been linked to elevated WNT ligand expression in PDAC patient–derived organoids (22), we observed no significant changes in *GATA6* expression in KPTN cells, suggesting alternative regulatory mechanisms for *WNT* upregulation. In addition, it has been reported that *NF2* overexpression in PDAC cell lines is associated with attenuation in the WNT/β-catenin signaling, which is consistent with the WNT activation observed in our *NF2*-KO model (49). However, the previous observation was linked to the ability of *NF2* to disrupt FOXM1-mediated β-catenin nuclear translocation, whereas we report a direct induction of autonomous WNT7B ligand secretion in pancreatic acinar cells upon *NF2* KO, representing a distinct mechanism of *WNT* signaling regulation during early PDAC progression.

There have been concerns as to what extent organoid culture systems can recapitulate the TME in cancer research. It was reported that a state shift in PDAC organoids can be achieved by simply manipulating culture medium components (45), highlighting the flexibility of experimental organoid models. Our data showed that organoid culture can serve as a complementary system to capture intrinsic transcriptomic predisposition driven by genetic alterations. By comparing our in vitro and in vivo screen outcomes as well as RNA-Seq data, we demonstrated marked upregulation of genes involved in the cellular response to starvation in tumor cells, whereas higher expression of genes responsible for glucose and amino acid metabolism occurred in organoids. These findings allowed us to establish a modified LM to mimic in vivo condition, which revealed enhanced activation of macropinocytosis by *NF2* inactivation under nutrient stress. Alterations in other cellular programs were also observed between in vivo tumors and in vitro cultures, including epithelial-mesenchymal transition, ECM organization, focal adhesion, and collagen formation. These differences likely reflect the distinct environmental contexts of in vivo tumor cells versus organoid culture models and warrant further investigation.

Cancer drug resistance remains a major obstacle to achieving long-term therapeutic success, and is driven by a complex interplay of genetic, epigenetic, and microenvironmental factors. In this study, we observed that an increased driver mutation burden in engineered acinar cells led to enhanced resistance to a pan-RAS inhibitor, underscoring the role of genetic predisposition in drug resistance. Furthermore, we demonstrated that KPTN organoids adapted to low-nutrient conditions and exhibited resistance to multiple drugs, suggesting that nutrient stress induces a phenotypic shift that exacerbates therapeutic resistance. While many drugs show efficacy against cancer cells and xenograft tumors in preclinical models, they often fail in patients (51), probably because of the lack of a fully recapitulated TME and in vivo–like conditions. Our model system addresses this limitation by providing a robust platform to mimic in vivo settings. Together, our results emphasize the interplay between genetic alterations and environmental stress in driving therapeutic resistance and suggest that targeting both intrinsic and extrinsic pathways may be necessary to overcome resistance in PDAC.

Previous studies in mice have demonstrated that PDAC derived from different cells of origin may undergo distinct pathological mechanisms (2, 3). While the present study is focused on pancreatic acinar cells, we will take advantage of our unique human PDAC model in future studies to investigate PDAC progression in the ductal lineage. In addition, one limitation of this work is that all the xenograft tumors were generated subcutaneously in immunodeficient mice for efficient screening, which cannot recapitulate the full PDAC microenvironment spectrum. To address this, we will consider using humanized mouse models in future studies to enable investigation with the presence of immune components.

In our study, we chose not to include negative controls in the CRISPR library design in order to maintain a compact library size suitable for primary culture delivery constraints. This design decision was based on the assumption supported by prior CRISPR screen analyses and the MAGeCK algorithm based on the finding that most sgRNAs do not exhibit strong selection effects, allowing effective normalization even without negative controls. Consistent with this rationale, several previously published studies (52, 53) involving in vivo CRISPR screens have also excluded negative controls, supporting the feasibility of this alternative approach. Nevertheless, omitting negative controls may reduce the statistical power for FDR estimation and increase the likelihood of false-positives, particularly for sgRNAs with modest phenotypic effects. While this limitation does not affect our main conclusion regarding the role of *NF2*, further experimental validations are required to confirm other screen hits identified in the present study.

In summary, our system combining a 3D organoid culture of primary human pancreatic cells with a CRISPR screening technique serves as an effective platform to identify additional genes important for PDAC progression. By using this platform, we identified *NF2* inactivation as a potent driver for human pancreatic acinar transformation, promoting the aggressive progression of acinar-derived PDAC by inducing intrinsic changes to prime transformed cells for enhanced cell fitness under nutrient deprivation and therapeutic stress. We also identified a list of previously underappreciated tumor suppressor mutations which may provide a selective growth advantage during acinar transformation, warranting further investigation. The discovery of additional drivers in early PDAC development will provide opportunities to explore effective treatment strategies.

## Methods

### Sex as a biological variable.

For experiments involving human pancreatic tissues, the sex of organ donors is reported in Supplemental Table 1 and was not considered as a biological variable. The tissues used in this study were selected on the basis of the availability of resources to provide biological replicates for sufficient statistical power.

For animal experiments, we examined male and female mice, and similar findings are reported for both sexes. The animal’s sex was not considered in the study design, as no evidence exists to date for the effect of sex on the subcutaneous tumorigenesis of pancreatic cancer cells.

### Isolation of human primary pancreatic acinar cells.

Human islet–depleted pancreatic exocrine cell fractions were purchased from Prodo Laboratories. The cells were freshly collected from organ donors who had died as a result of acute trauma or anoxia (Supplemental Table 1) and shipped overnight to UT Health San Antonio. The pancreatic acinar cells were isolated by flow sorting as previously described (14). Briefly, exocrine tissue cells were incubated with FITC-conjugated UEA-1 (0.25 μg/mL, Vector Laboratories, FL-1061-5) for 10 minutes at 4°C. After washing with PBS, the cells were digested by incubation with TrypLE Express (Life Technologies, Thermo Fisher Scientific, 12605-028) for 5–8 minutes at 37°C. Cells were collected by centrifugation and washed with FACS buffer (10 mM EGTA, 2% FBS in PBS). Cells were then stained with Pacific blue–conjugated anti-CLA (BioLegend, 321308) and anti-7AAD (BioLegend, 420404) for 15 minutes at 4°C. Cell pellets were collected by centrifugation and washed with PBS. Flow sorting was performed using the FACSAria II (BD Biosciences), and acinar cells were collected in 100% FBS. The collected cells were washed with serum-free Advanced DMEM/F-12 media (Life Technologies, Thermo Fisher Scientific, 12634-010) for further processing.

### 3D organoid culture and chemical treatment.

Approximately 0.5 × 10^6^ freshly sorted acinar cells were resuspended in 10 μL ice-cold Type 2 Cultrex RGF Basement Membrane Extract (BME) (R&D Systems, 3533-010-02P) and then placed in the bottom of a prewarmed 24-well plate. After solidification at 37°C for 10 minutes, 500 μL organoid growth media were added to the well. The media was composed of serum-free advanced DMEM/F-12 media (Life Technologies, Thermo Fisher Scientific, 12634-010) supplemented with WNT3A conditioned medium (50%), recombinant human R-spondin 1 (500 ng/mL, R&D Systems, 4645-RS), noggin (200 ng/mL, R&D Systems, 6057-NG), FGF10 (100 ng/mL, R&D Systems, 345-FG), EGF (50 ng/mL, R&D Systems, 236-EG), PGE II (1 nM, Thermo Fisher Scientific, 22-961-0), A83-01 (0.5 μM, R&D Systems, 2939), nicotinamide (10 mM, Thermo Fisher Scientific, 48-190-7100GM), penicillin/streptomycin (1 mM, MilliporeSigma, P4333), HEPES (10 mM, Life Technologies, Thermo Fisher Scientific, 15630), and GlutaMAX 1× (Life Technologies, Thermo Fisher Scientific, 35050).

Modified organoid growth media were prepared as described below and used as indicated in Results. The CM was prepared similarly as organoid growth media, except without WNT3A supplement. The LM was prepared by supplementing basic DMEM (no glucose, no glutamine, Thermo Fisher Scientific, A1443001) with 5% CM. For drug response tests, all the chemicals were prepared as stock solutions, followed by dilution in the appropriate culture medium to reach final working concentrations. The chemicals used in this study included LGK974 (MedChemExpress, HY-17545), EIPA (MedChemExpress, HY-101840), FRAX597 (MedChemExpress, HY-15542A), RMC-7977 (Chemgood, C-1010), gemcitabine (Thermo Fisher Scientific, AC461060010), and WH244 (a gift from Daohong Zhou, Department of Biochemistry and Structural Biology, The University of Texas Health San Antonio, San Antonio, Texas, USA) (48). Organoid images after chemical treatment were captured using an EVOS XL Core or Leica DMI6000 B microscope.

### Genetic engineering human primary pancreatic acinar cells.

Introduction of oncogenic *KRAS^G12V^* and CRISPR KO of *CDKN2A/p16* and *TP53* were performed as described previously(15). Briefly, a plasmid expressing *KRAS^G12V^* cDNA with mCherry was a gift from Seung Kim (Stanford University) (54). CRISPR gRNAs targeting *CDKN2A/p16* and *TP53* were subcloned into lentiCRISPR v2 vector (Addgene, 52961). CRISPR gRNA targeting *NF2* was subcloned into lentiGuide-Hygro-mTagBFP2 vector (Addgene, 99374). Lentiviruses containing these constructs were packaged in 293T cells by cotransfection with packaging plasmids pMD2.G (Addgene, 12259) and psPAX2 (Addgene, 12260). The primary acinar cells were transduced with lentivirus containing each construct with supplementation of polybrene at 10 μg/mL. The transduced cells were then subjected to antibiotic and/or functional selections. To select *KRAS^G12V^*-expressing cells, G418 at 1,000 μg/mL was added to the organoid culture medium. For the selection of CRISPR-*CDKN2A/p16* transduction, cells were treated with puromycin (1 μg/mL). For the selection of *TP53* KO, 10 μM nutlin-3 was added to the culture medium. To select *NF2*-KO cells, hygromycin at 300 μg/mL was added to the culture medium. The expression of mCherry in the *KRAS^G12V^* cassette was visualized under a Leica DMI6000 B fluorescence microscope. *CDKN2A/p16* and *TP53* mutations were confirmed by Sanger sequencing. KO of *NF2* at the protein level was verified by Western blotting.

The sgRNA and primer sequences were as follows: *CDKN2A/p16* sgRNA, GGCTGGCCACGGCCGCGGCC; *TP53* sgRNA, ACTTCCTGAAAACAACGTTC; *NF2* sgRNA, GCTTGGTACGCAGAGCACCG; *CDKN2A/p16* mutation genotyping forward primer, CGGTCCCTCCAGAGGATTTG; *CDKN2A/p16* mutation genotyping reverse primer, TGGAGGCTAAGTAGTCCCAG; *TP53* mutation genotyping forward primer, TGCTGGATCCCCACTTTTCC; and *TP53* mutation genotyping reverse primer, GGATACGGCCAGGCATTGAA.

### CRISPR-KO library preparation.

From publicly available data, we compiled a list of 199 recurrently mutated potential tumor suppressor genes reported in clinical PDAC patients (7–13). A CRISPR-KO sgRNA library was designed to target these potential tumor suppressors containing a total of 796 sgRNAs with 4 sgRNAs for each target gene (Supplemental Table 2). The pooled sgRNA oligonucleotides were purchased from Twist Bioscience. After PCR amplification, pooled sgRNAs were cloned into lentiGuide-Hygro-mTagBFP2 vector (Addgene, 99374) by enzyme digestion followed by ligation using NEBuilder HiFi DNA Assembly (New England BioLabs, E2621S). Electroporation was then performed using 100 ng assembly products to transform 50 μL Endura electrocompetent cells at 1,800 V, 25 μF, 200 ohm using a Bio-Rad Gene Pulser. After recovery in 2 mL recovery medium for 1 hour, the transformed bacteria cells were plated in a total of 60 agar plates (100 mm) and incubated at 30°C overnight. Approximately 1.6 × 10^6^ colonies were harvested from all the plates, which gave approximately 200 coverage for each individual sgRNA. Plasmid DNA was then extracted using GeneJet Midi prep and subjected to NGS to validate the distribution of each individual sgRNA in the library. The plasmid DNA was used to transfect 293T cells using Lipofectamine 3000 reagent (Thermo Fisher Scientific) together with the packaging plasmids pMD2.G and psPAX2 for lentivirus production.

### CRISPR-KO screen and data analysis.

The pancreatic acinar organoids were transduced with lentivirus containing the CRISPR sgRNA library at a low MOI of approximately 0.3, followed by antibiotic selection with hygromycin at 300 μg/mL. Successful transduction was verified by confirming the cellular expression of the blue fluorescent protein tag from the vector construct using a Leica DMI6000 B fluorescence microscope. The transduced cells were expanded for 1 passage, and a fraction of the cells was collected to serve as a reference control. For the in vivo screen, the expanded cells were subcutaneously transplanted into the hind flank of NSG mice. The xenograft tumor tissues were collected after 8 weeks, followed by DNA extraction using a Genomic DNA Clean & Concentrator 25 kit (Zymo Research, D4065). For the in vitro screen, the expanded cells were continuously passaged for 8 weeks (7–9 passages), followed by extraction of cellular DNA. The collected DNA containing sgRNA library sequences was subject to a 2-step PCR protocol for library amplification and sample indexing, followed by NGS analysis at the Greehey Children’s Cancer Research Institute (GCCRI) Genome Sequencing Facility at UT Health San Antonio. The screen was performed using 4 independently established acinar cultures, with 2 replicates of in vivo tumor samples and 1 replicate of in vitro culture sample for each. The count summary and statistical analysis of sgRNA enrichment from fastq data were performed using MAGeCK software v0.5.9.5 (55). A significant positive enrichment at the target gene level was considered when the fold change was greater than 1 and the *P* value was less than 0.05 compared with the corresponding reference control sample.

### 3D organoid cell viability assay.

Cell viability of 3D organoid cultures in different culture conditions and/or after chemical treatment was assessed using the CellTiter-Glo 3D Cell Viability Assay Kit (Promega) according to the manufacturer’s instructions. Briefly, 8,000 cells were resuspended in 5 μL ice-cold BME and placed in the center of each well in a 96-well plate. After solidification at 37°C for 10 minutes, 100 μL organoid growth media were added to each well. After 3 days of incubation, the medium was refreshed with supplementation of different chemicals as indicated in Results. At the endpoint, 100 μL CellTiter-Glo 3D Cell Viability reagent (Promega) was added to each well, followed by vigorous mixing to induce cell lysis. The plate was placed at room temperature for 25 minutes and subjected to luminescence detection using a BioTek Synergy H1 microplate reader.

### Flow cytometric analysis for BFP and FITC-dextran.

KPT and KPTN cells were mixed at a 1:1 ratio and embedded in BME for 3D organoid culture. A fraction of the mixed cells was collected at passage 1 to passage 7 for flow cytometric analysis of the BFP signal inserted into the *NF2* sgRNA construct using a BD LSR II flow cytometer. KPT and KPTN monocultures were subject to the same analysis as the controls.

For dextran uptake analysis, KPT and KPTN cells were cultured in CM for 4 days to allow organoid formation. On day 4, the organoids were exposed to low-nutrient medium for 1 day to induce starvation responses. A control group was maintained in fresh complete nutrient medium. Half of the medium (~200 μL) from each well was collected, and FITC-conjugated dextran 70 (Thermo Fisher Scientific, D1822) was added to reach a final concentration of 1 mg/mL. Dispase was added to the remaining medium (2 U/mL) and incubated at 37°C for 20 minutes to release organoids from the BME. The organoids were then collected by centrifugation at 200*g* for 3 minutes. Half of the organoids were kept as a baseline control, while the other half was resuspended in dextran 70–containing medium and incubated at 37°C for 30 minutes. The organoids were then washed twice with PBS and dissociated into single cells for flow cytometric analysis using a BD LSR II flow cytometer.

### Detection of macropinocytosis in Panc1 cells with NF2 overexpression.

Merlin-overexpressing Panc1 cells were established by transfecting parental Panc1 cells with pcDNA3 merlin plasmids (Addgene, 11623) followed by G418 selection (1,000 μg/mL). Cells transfected with an empty vector served as a control. To induce starvation responses, the cells were cultured in a 6-well plate in DMEM with 10% FBS for 3 days and then exposed to LM (0% FBS) for 1 day. FITC-conjugated dextran 70 (Thermo Fisher Scientific, D1822) was then added to each well at a final concentration of 1 mg/mL, followed by a 30-minute incubation at 37°C. The FITC signal was visualized under a Leica DMI6000 B fluorescence microscope.

### Animal experiments.

The maximal xenograft tumor size in each mouse did not exceed 2 cm at the largest diameter, as permitted by institutional guidelines. For all the animal experiments, 6- to 8-week-old female and male NSG (NOD.Cg-*Prkdc^scid^ IL2Rg^tm1Wjl^*/SzJ) mice were used (purchased from The Jackson Laboratory, strain 005557). The mice were housed at a maximum of 5 per cage in a pathogen-free system with ad libitum access to water and food and a 12-hour light/12-hour dark cycle at 20°C–25°C and 50%–60% humidity. Details on the study design and sample size for the animal studies are described below and were determined on the basis of the availability of resources to provide at least 4 biological replicates for sufficient statistical power. No criteria were used for inclusion or exclusion of animals during the experiments, and there were no exclusions of any animals or data points in the data analysis. Randomization was not done in this study, and confounders were not controlled for. When comparing samples from different groups, we used paired samples from the same culture origin and made sure to include multiple biological replicates to minimize potential heterogeneity. The authors who were responsible for animal experiments as well as following data analysis were aware of the group allocation at all stages of the experiment.

For in vivo CRISPR screen experiments, details on the study design are described above in *CRISPR-KO screen and data analysis*. Briefly, a total of 8 NSG mice were subjected to xenograft transplantation (4 independently established acinar cultures, with 2 replicates each). Tumor xenografts were established by subcutaneously injecting approximately 1 × 10^6^ engineered cells (suspended in 100 μL of 50% BME/50% Advanced DMEM/F-12) into the hind flank of mice. The xenograft tumor tissues were collected after 8 weeks, followed by DNA extraction and then NGS analysis. The enrichment of any sgRNA in tumor tissue DNA was identified by comparison with the paired control as described previously. For functional verification of the effect from *NF2* KO on tumor development, a total of 45 mice were subjected to subcutaneous transplantation of approximately 1 × 10^6^ engineered cells (*n* = 4–7 for each of the 4 independent cultures and each of the 2 genotypes, as indicated in Figure 2C). After 8 weeks, the mice were euthanized to collect the tumor tissues for further histological analysis. The difference in tumor size was compared between KPTN tumors and KPT counterparts from the same culture origin. Tumor volume was calculated as follows: V = L × W^2^/2 (V, volume; L, length, W, width). To assess the effect of LGK974 on KPTN tumor growth, a total of 8 mice were transplanted with approximately 1 × 10^6^ KPTN cells on both sides of hind flank. After 1 month to allow tumor development, tumor-bearing mice were separated into 2 groups (*n* = 4 mice for each group) and received a daily dose of LGK974 (10 mg/kg, oral gavage) or vehicle control for 40 days. Tumor size and mouse body weight were recorded every 5 days. At the endpoint, the mice were euthanized to collect tumor tissues for further analysis.

### Western blot analysis.

Protein expression in the in vitro cell culture was detected by Western blotting following a standard protocol. Briefly, the cells were harvested to collect cell lysates. Approximately 20 μg protein from cell lysates was loaded into each lane of a 10% SDS-PAGE gel followed by electrophoresis at 90 V. The proteins on the gel were then transferred onto a PVDF membrane at a constant voltage of 80 V for 1.5 hours on ice. After transfer, the membranes were incubated in blocking solution (3% BSA in TBST) with continuous rocking for 60 minutes and then incubated with a primary antibody overnight at 4°C. The membranes were then washed and incubated with an HRP-conjugated secondary antibody for 2 hours at room temperature. The membranes were subsequently washed and incubated with ECL Western blot substrates for 2 minutes, followed by signal capture using an Amersham Imager 600. The following primary and secondary antibodies were used: rabbit anti–human NF2 (Abclonal, A0739), dilution 1:500; mouse anti–human KRAS (MilliporeSigma, OP24), dilution 1:100; mouse anti–human p53 (Santa Cruz Biotechnology, sc-126), dilution 1:250; mouse anti–human GAPDH (Santa Cruz Biotechnology, sc-32233), dilution 1:500; mouse anti–rabbit IgG-HRP (Santa Cruz Biotechnology, sc-2357), dilution 1:1,000; goat anti–mouse IgG-HRP (Santa Cruz Biotechnology, sc-2005), dilution 1:1,000.

### H&E and immunofluorescence staining.

Freshly collected xenograft tumor tissues were fixed to prepare a paraffin-embedded block. Tissue sectioning H&E and alcian blue staining were performed at the histology laboratory at UT Health San Antonio following standard protocols. For immunofluorescence staining, paraffin-embedded tissue section were deparaffinized, rehydrated, and submerged in 200°C heated R-Universal Epitope Recovery Buffer solution (Electron Microscopy Sciences, AP0530) for 30 minutes. After sitting at room temperature for 20 minutes, sections were permeabilized using 0.5% PBST (0.5% Triton X-100 in PBS) for 10 minutes and blocked with 5% donkey serum in 0.1% PBST for 60 minutes at room temperature. Sections were then incubated with primary antibodies diluted in blocking solution at 4°C overnight. Sections were subsequently incubated with fluorescence-tagged Alexa Fluor secondary antibodies diluted in 5% blocking solution for 1 hour at room temperature. Additionally, sections were incubated with DAPI (1:1,000, Invitrogen, Thermo Fisher Scientific, P36935) for 4 minutes at room temperature. Finally, sections were covered with a drop of VectaShield Vibrance Antifade Mounting Medium (Vector Laboratories, H-1700). All histology and fluorescence images were captured using a Leica DMI6000 B microscope and compatible software (Leica Microsystems). Fluorescence signal intensities were quantified using ImageJ v1.5g software (NIH).

The following primary and secondary antibodies were used: mouse anti–human STEM121 (Takara Bio, Y40410, dilution 1:200); mouse anti–human/–mouse/–rat KRT19 (DSHB, Troma-III, dilution 1:50); mouse anti–human/–mouse/–rat YAP (63.7) (Santa Cruz Biotechnology, sc-101199, dilution 1:50); and Alexa Fluor 488–conjugated AffiniPure Donkey Anti–Mouse IgG (H+L) (Jackson ImmunoResearch, 715-545-150, dilution 1:250).

### Bulk RNA-Seq library preparation and data analysis.

Cell pellets of cultured organoids were collected and stored in TRIzol (Zymo Research, catalog R2050-1-200) for total RNA extraction using Zymo Direct-zol RNA Miniprep Kit (Zymo Research, R2063) following the manufacturer’s instructions. Tumor tissues were flash-frozen in liquid nitrogen and ground into fine powder with the addition of TRIzol, followed by RNA extraction using the Zymo Direct-zol RNA Miniprep Kit. Indexed cDNA libraries were prepared using the Illumina Stranded mRNA Prep Ligation kit (Illumina, 20040532) following the manufacturer’s instructions. The cDNA libraries were then submitted to the GCCRI Genome Sequencing Facility at UT Health San Antonio for high-throughput sequencing analysis using the Illumina HiSeq 3000 or NovaSeq 6000 System.

Raw sequencing data were aligned to the reference genome GRCh38 or GRCm38 using TopHat 2.1.1. Gene expression reads were quantified using HTSeq 0.11.1. For tumor tissue samples, in order to separate human and mouse transcripts, the raw reads were aligned to a concatenated human and mouse genome. Reads for which multiple alignments shared the top score were discarded to remove those aligned to both the human and mouse genome (56). Differential expression analysis of the read counts from aligned RNA-Seq data was performed using the DESeq2 1.36.0 package in R software. A prefiltering process was applied for each analysis to remove genes with a low read count, i.e., we only retained genes with more than 10 reads in at least half of the samples of at least 1 comparison group. The DEGs were defined as those with a fold change of greater than 2, and a Benjamin-Hochberg–adjusted (BH-adjusted) *P* value of less than 0.05. Gene expression heatmaps were generated using the *z* score of the transformed/normalized read count as indicated in each figure. Overrepresentation analysis for the lists of genes was performed using the clusterProfiler 4.4.4 package in R with default settings. A significant enrichment was considered with a multiple test–adjusted *P* value of less than 0.05.

### scRNA-Seq library preparation and data analysis.

scRNA-Seq samples were prepared using the Evercode Fixation V2 kit and the Evercode WT V2 Kit (Parse Biosciences) following the manufacturer’s instructions. Briefly, organoid culture or tumor tissues were dissociated into single cells by enzyme digestion and flow sorting. The single cells were subjected to a fixation procedure using the Evercode Fixation V2 kit, followed by a split-pool combinatorial barcoding protocol in 96-well format using the Evercode WT V2 Kit. The barcoded cDNAs were equally dispersed into 8 indexed sublibrairies and were submitted for high-throughput sequencing analysis using the Illumina NovaSeq X plus System.

Processing of the raw sequencing reads including alignment, demultiplexing, and separation of human and mouse reads in the scRNA-Seq data was performed using ParseBiosciences-Pipeline software v1.1.2, which was designed to be compatible with the sample preparation protocol. The reference genomes GRCh38 and GRCm38 were used for alignment. The subsequent analysis was performed using Seurat v.5.0.1 in R software. A quality control step was performed to remove low-quality cells, including possible cell debris (nFeature <200), cell doublets (nFeature >9,000 for organoid samples and nFeature >5,000 for tumor samples), and cells with excessive cellular stress (>15% human mitochondrial gene or >10% mouse mitochondrial gene expression). The filtered Seurat object was subjected to a series of standard Seurat commands to identify the different cell populations present in the dataset. For in vitro organoid samples, an extra “harmony integration” step was performed before downstream analysis to remove batch effects that were probably introduced during sample preparation. For in vivo tumor samples, the combined Seurat object was subsetted to contain human or mouse cells only for downstream analysis. Expression scores of a list of gene signatures were calculated using the “AddModuleScore” command in Seurat. Single-cell trajectory analysis was performed using Monocle3 v.1.3.4 in R software with the default setting. The Seurat object containing all organoid cells were passed into Monocle3 to infer their cell-type transition states, by setting cluster 1 as the root. Cell-cell communication analysis was performed using CellChat v2.1.2 in R software with the default setting.

### Kaplan-Meier plot of TCGA data.

Clinical prognosis of gene expression was assessed using TCGA PDAC patient data. For prognosis analysis of a single gene, the patients were grouped by median gene expression using RNA-Seq data, followed by Kaplan-Meier plotting with survival data. For prognosis of a group of genes, the gene signature expression score for each patient was calculated using GSVA v1.52.3 from their RNA-Seq data (57). Then, the patients were grouped by median expression score followed by Kaplan-Meier plotting. The Kaplan-Meier plot was generated using survival v3.7 in R environment.

### Protein-protein interaction network analysis.

Protein-protein interaction analysis of *NF2* was performed using STRING PubMed query and visualized in Cytoscape v3.10.3 software with the default setting.

### Statistics.

Statistical analyses of CRISPR screen enrichment were performed using the MAGeCK algorithm. All the bioinformatics analyses of high-throughput RNA-Seq data were performed using the R packages described in Methods and the figure legends. Quantitative analysis of experimental results between 2 groups was performed using a 2-tailed Student’s *t* test. For experiments with more than 2 groups, the data were first subject to 1-way ANOVA to determine if there are any significant differences between the means of the groups, followed by multiple pairwise-comparison using Tukey’s honest significant differences test. The sample size in each experiment is described in Results and the figure legends. A P value of less than 0.05 was considered significant. Data in all the bar figures are presented as mean ± SD.

### Study approval.

All experiments in this study using primary human pancreatic tissues were reviewed by the UT Health San Antonio IRB. The patient donors were deidentified, with available information on sex, race, age, weight, height, and cause of death. The IRB committee agreed that this study does not require IRB approval because it is either not human research as defined by Department of Health and Human Services (HHS) regulations at 45 Code of Federal Regulation (CFR) 46 and FDA regulations at 21 CFR 56, and the project does not include nonroutine intervention or interaction with a living individual for the primary purpose of obtaining data regarding the effect of the intervention or interaction, nor did the researchers obtain private, identifiable information about living individuals.

All animal experiment protocols were approved by the IACUC of UT Health San Antonio (protocol no. 20130023AR) and were performed in accordance with relevant guidelines and regulations.

### Data availability.

A Supporting Data Values file is provided that includes all data represented in graphs as the mean ± SD. The raw and processed high-throughput sequencing data generated in this study have been deposited in the Gene Expression Omnibus (GEO) database (GSE292511, GSE292512, and GSE292513). TCGA pancreatic cancer patient data are available in the NCI GDC data portal (https://portal.gdc.cancer.gov/projects/TCGA-PAAD). The human reference genome GRCh38 and mouse reference genome GRCm38 are available in the UCSC Genome Browser (https://genome.ucsc.edu/index.html).

## Author contributions

PW and JL conceived the study. YX, MHN, CH, JL, and PW were responsible for data curation. YX, MHN, JL, AAD, CH, FES, SK, DZ, HX, YL, and PW conducted formal analysis. PW acquired funding. YX, MHN, and JL designed the methodology. PW and JL were responsible for project administration. PW supervised the study. YX, JL, and PW wrote the original draft of the manuscript. YX, MHN, DZ, LZ, YL, JL, and PW reviewed and edited the manuscript.

## Funding support

This work is the result of NIH funding, in whole or in part, and is subject to the NIH Public Access Policy. Through acceptance of this federal funding, the NIH has been given a right to make the work publicly available in PubMed Central. The funders had no role in study design, data collection and analysis, decision to publish, or preparation of the manuscript.

- Cancer Prevention and Research Institute of Texas (R1219,
- to PW).
- National Cancer Institute (NCI)/NIH (R21 CA218968 and R01 CA237159, to PW).
- (NIDDK), NIH (R01 DK110361, to PW).
- William and Ella Owens Medical Research Foundation
- (to PW).
- PW is a Cancer Prevention and Research Institute of Texas (CPRIT) scholar.
- The University of Texas Health Science Center San Antonio, T32CA148724 and T32GM148752 (to AD).
- Part of data were generated at the Flow Cytometry Shared Resource at UT Health San Antonio, which is supported by the NCI (P30CA054174, to the Mays Cancer Center); CPRIT (RP210126); and the NIH (1S10OD030432-01A1).
- High-throughput sequencing data described in this study were generated in the Genome Sequencing Facility/Mays Cancer Center Next-generation Sequencing Facility, which is supported by the NCI, NIH (P30 CA054174, to UT Health San Antonio, Cancer Center at UT Health San Antonio); NIH Shared Instrument grant (S10OD030311, S10 grant for the NovaSeq 6000 System); and CPRIT Core Facility Award (RP220662).

## Supplementary Material

Supplemental data

Unedited blot and gel images

Supplemental table 1

Supplemental table 2

Supplemental table 3

Supplemental table 4

Supplemental table 5

Supplemental table 6

Supplemental table 7

Supporting data values

## Version 1 01/02/2026

Electronic publication
