# Supplementary material for: NF2 loss malignantly transforms human pancreatic acinar cells and enhances cell fitness under environmental stress
Source: J Clin Invest. 2026 Jan 2;136(1):e194395. doi: 10.1172/JCI194395 (PMC12721884; doi:10.1172/JCI194395)
Supplement: Supplemental data [file jci-136-194395-s118.pdf]

Supplementary Materials for

NF2 loss malignantly transforms human pancreatic acinar cells and enhances cell fitness under environmental stress

Yi Xu *et al.*

*Corresponding author. Email: Wangp3@uthscsa.edu, liuj8@uthscsa.edu

This PDF file includes:

- Figure. S1 to S8 and corresponding legends

- Description of Supplementary Tables 1-7

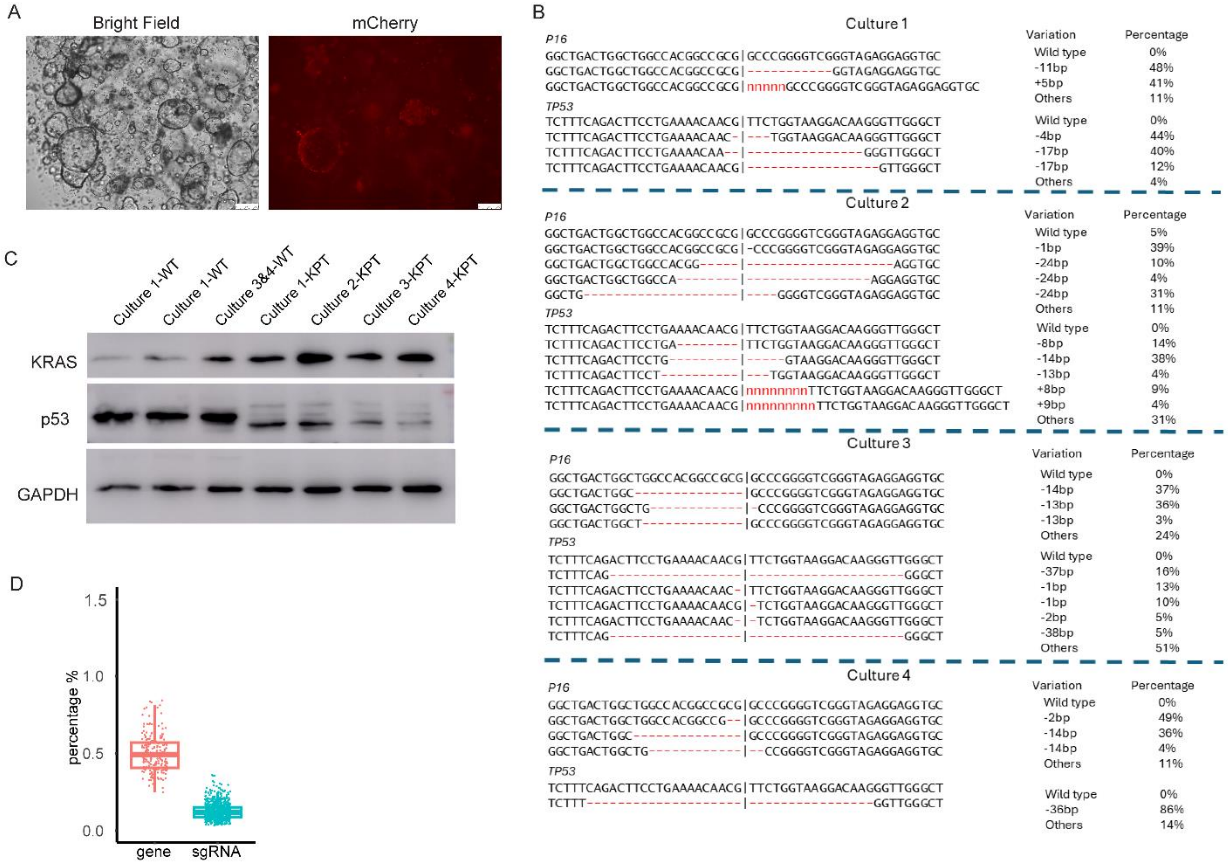
A
Bright Field
mCherry
B
Culture 1
P16
Variation
Percentage
GGCTGACTGGCTGGCCACGGCCGCG|GCCCGGGGTCGGGTAGAGGAGGTGC
Wild type 0%
GGCTGACTGGCTGGCCACGGCCGCG|-----------GGTAGAGGAGGTGC
-11bp 48%
GGCTGACTGGCTGGCCACGGCCGCG|nnnnnGCCCGGGGTCGGGTAGAGGAGGTGC
+5bp 41%
Others 11%
TP53
TCTTTCAGACTTCCTGAAAACAACG|TTCTGGTAAGGACAAGGGTTGGGCT
Wild type 0%
TCTTTCAGACTTCCTGAAAACAAC-|---TGGTAAGGACAAGGGTTGGGCT
-4bp 44%
TCTTTCAGACTTCCTGAAAACAA--|---------------GGGTTGGGCT
-17bp 40%
TCTTTCAGACTTCCTGAAAACAACG|-----------------GTTGGGCT
-17bp 12%
Others 4%
Culture 2
P16
Variation
Percentage
GGCTGACTGGCTGGCCACGGCCGCG|GCCCGGGGTCGGGTAGAGGAGGTGC
Wild type 5%
GGCTGACTGGCTGGCCACGGCCGCG|-CCCGGGGTCGGGTAGAGGAGGTGC
-1bp 39%
GGCTGACTGGCTGGCCACGG-----|-------------------AGGTGC
-24bp 10%
GGCTGACTGGCTGGCCA--------|---------------AGGAGGTGC
-24bp 4%
GGCTG--------------------|----GGGGTCGGGTAGAGGAGGTGC
-24bp 31%
Others 11%
TP53
TCTTTCAGACTTCCTGAAAACAACG|TTCTGGTAAGGACAAGGGTTGGGCT
Wild type 0%
TCTTTCAGACTTCCTGA--------|TTCTGGTAAGGACAAGGGTTGGGCT
-8bp 14%
TCTTTCAGACTTCCTG---------|-----GTAAGGACAAGGGTTGGGCT
-14bp 38%
TCTTTCAGACTTCCT----------|---TGGTAAGGACAAGGGTTGGGCT
-13bp 4%
TCTTTCAGACTTCCTGAAAACAACG|nnnnnnnnTTCTGGTAAGGACAAGGGTTGGGCT
+8bp 9%
TCTTTCAGACTTCCTGAAAACAACG|nnnnnnnnnTTCTGGTAAGGACAAGGGTTGGGCT
+9bp 4%
Others 31%
Culture 3
P16
Variation
Percentage
GGCTGACTGGCTGGCCACGGCCGCG|GCCCGGGGTCGGGTAGAGGAGGTGC
Wild type 0%
GGCTGACTGGC--------------|GCCCGGGGTCGGGTAGAGGAGGTGC
-14bp 37%
GGCTGACTGGCTG------------|-CCCGGGGTCGGGTAGAGGAGGTGC
-13bp 36%
GGCTGACTGGCT-------------|GCCCGGGGTCGGGTAGAGGAGGTGC
-13bp 3%
Others 24%
TP53
TCTTTCAGACTTCCTGAAAACAACG|TTCTGGTAAGGACAAGGGTTGGGCT
Wild type 0%
TCTTTCAG-----------------|---------------------GGGCT
-37bp 16%
TCTTTCAGACTTCCTGAAAACAAC-|TTCTGGTAAGGACAAGGGTTGGGCT
-1bp 13%
TCTTTCAGACTTCCTGAAAACAACG|-TCTGGTAAGGACAAGGGTTGGGCT
-1bp 10%
TCTTTCAGACTTCCTGAAAACAAC-|-TCTGGTAAGGACAAGGGTTGGGCT
-2bp 5%
TCTTTCAG-----------------|---------------------GGGCT
-38bp 5%
Others 51%
Culture 4
P16
Variation
Percentage
GGCTGACTGGCTGGCCACGGCCGCG|GCCCGGGGTCGGGTAGAGGAGGTGC
Wild type 0%
GGCTGACTGGCTGGCCACGGCCG--|GCCCGGGGTCGGGTAGAGGAGGTGC
-2bp 49%
GGCTGACTGGC--------------|GCCCGGGGTCGGGTAGAGGAGGTGC
-14bp 36%
GGCTGACTGGCTG------------|--CCGGGGTCGGGTAGAGGAGGTGC
-14bp 4%
Others 11%
TP53
TCTTTCAGACTTCCTGAAAACAACG|TTCTGGTAAGGACAAGGGTTGGGCT
Wild type 0%
TCTTT--------------------|----------------GGTTGGGCT
-36bp 86%
Others 14%
C
Culture 1-WT
Culture 1-WT
Culture 3&4-WT
Culture 1-KPT
Culture 2-KPT
Culture 3-KPT
Culture 4-KPT
KRAS
p53
GAPDH
D
percentage %
1.5
1.0
0.5
0.0
gene
sgRNA

Figure S1. Establishing engineered acinar organoid culture and validating CRISPR sgRNA library. A. Representative bright field and fluorescence images of acinar organoids transduced with lentivirus expressing mCherry tagged *KRAS*^{G12V} construct from 4 biological replicates. Scale bar = 250 µm. **B.** Sanger sequencing analysis of *CDKN2A/p16* and *TP53* at the sgRNA target site in 4 independent cultures. **C.** Western blot of KRAS and p53 expression in wild type and KPT mutant organoids. **D.** Unbiased distribution of all 796 sgRNAs and corresponding target genes in the library plasmid DNA. The middle line of box represents the median value, the bounds of box represent the Interquartile Range, and the whiskers extend to 1.5 × Interquartile Range.

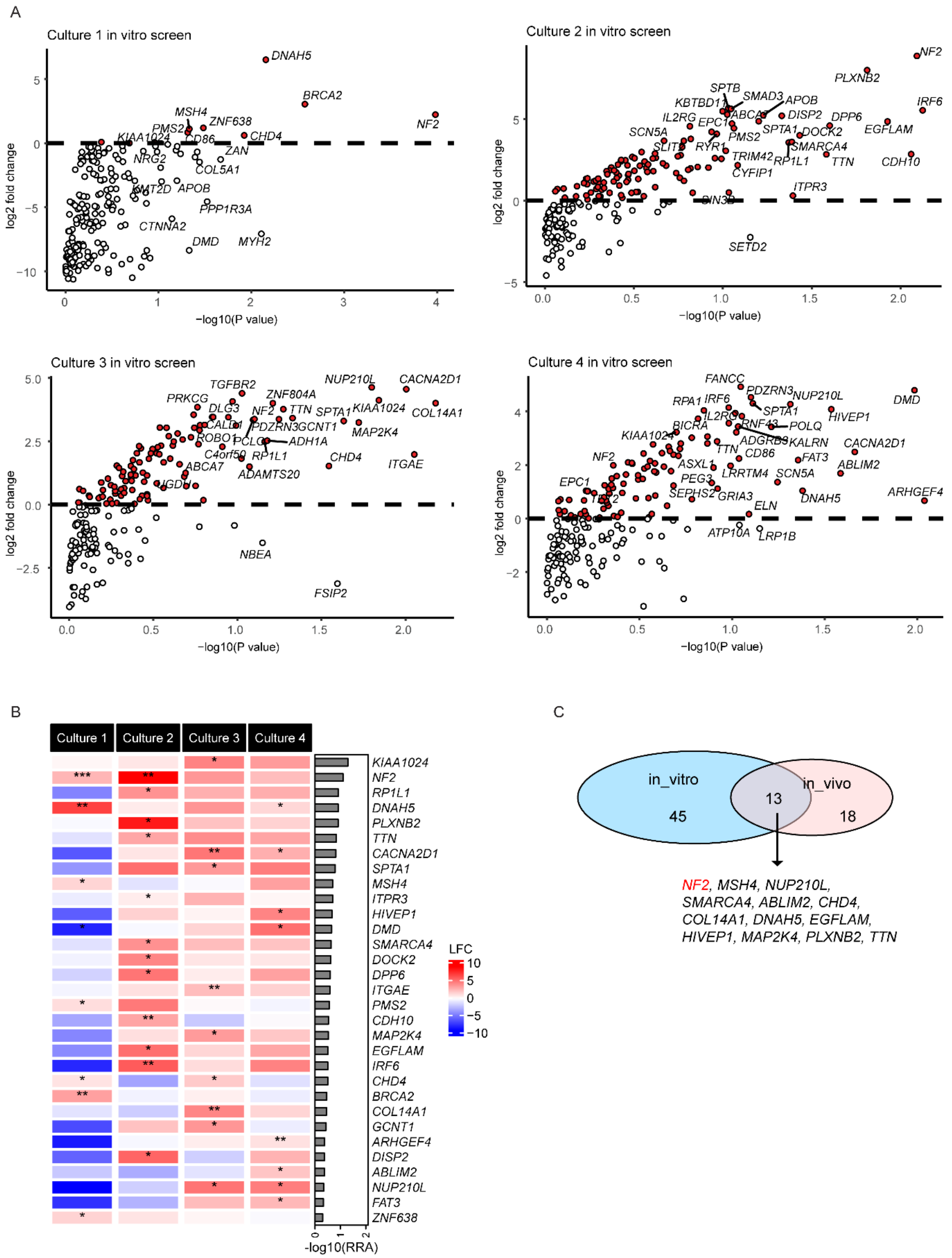

A
Culture 1 in vitro screen
log2 fold change
−log10(P value)
DNAH5
BRCA2
NF2
MSH4
ZNF638
PMS2
KIAA1024
CD86
CHD4
ZAN
NRG2
COL5A1
KMT2D
APOB
PPP1R3A
CTNNA2
DMD
MYH2
Culture 2 in vitro screen
NF2
PLXNB2
IRF6
SPTB
KBTBD11
SMAD3
APOB
IL2RG
EPC1
ABCA2
DISP2
DPP6
SCN5A
PMS2
SPTA1
DOCK2
EGFLAM
SLIT3
RYR1
SMARCA4
TRIM42
RP1L1
TTN
CDH10
CYFIP1
ITPR3
SIN3B
SETD2
Culture 3 in vitro screen
TGFBR2
NUP210L
CACNA2D1
PRKCG
ZNF804A
DLG3
NF2
TTN
SPTA1
KIAA1024
COL14A1
CALD1
PDZRN3
GCNT1
MAP2K4
ROBO1
PCLO
ADH1A
C4orf50
RP1L1
CHD4
ABCA7
ITGAE
ADAMTS20
NBEA
FSIP2
Culture 4 in vitro screen
FANCC
PDZRN3
NUP210L
DMD
RPA1
IRF6
SPTA1
IL2RG
HIVEP1
BICRA
RNF43
POLQ
KIAA1024
ADGRB3
KALRN
CACNA2D1
NF2
TTN
CD86
FAT3
ABLIM2
ASXL1
LRRTM4
SCN5A
EPC1
PEG3
SEPHS2
GRIA3
DNAH5
ARHGEF4
ELN
ATP10A
LRP1B
B
Culture 1
Culture 2
Culture 3
Culture 4
KIAA1024
NF2
RP1L1
DNAH5
PLXNB2
TTN
CACNA2D1
SPTA1
MSH4
ITPR3
HIVEP1
DMD
SMARCA4
DOCK2
DPP6
ITGAE
PMS2
CDH10
MAP2K4
EGFLAM
IRF6
CHD4
BRCA2
COL14A1
GCNT1
ARHGEF4
DISP2
ABLIM2
NUP210L
FAT3
ZNF638
-log10(RRA)
LFC
C
in_vitro
in_vivo
45
13
18
NF2, MSH4, NUP210L,
SMARCA4, ABLIM2, CHD4,
COL14A1, DNAH5, EGFLAM,
HIVEP1, MAP2K4, PLXNB2, TTN

Figure S2. ***In vitro*** **CRISPR screen results. A.** Scatter plot of the enrichment of target genes from *in vitro* CRISPR screen from 4 independent cultures. The Y axis represents log2 fold change of the sgRNA distribution of an indicated target gene compared with the control. X axis represents -log10 P value. Positively enriched targets (log2 fold change > 0) in individual replicate were highlighted in red. **B.** Heatmap of all target genes whose sgRNAs were found to be positively enriched (log2 fold change > 0, P value < 0.05) in any of the 4 independent cultures. The color scale represents log2 fold change (LFC) of sgRNA distribution compared with the control. The side bar plot indicates the averaged -log10 (RRA) value of each targe gene across all replicates. *, **, *** indicate p-value < 0.05, 0.01, 0.001, respectively. **C.** Comparison of the enriched target genes between *in vivo* and *in vitro* screen.

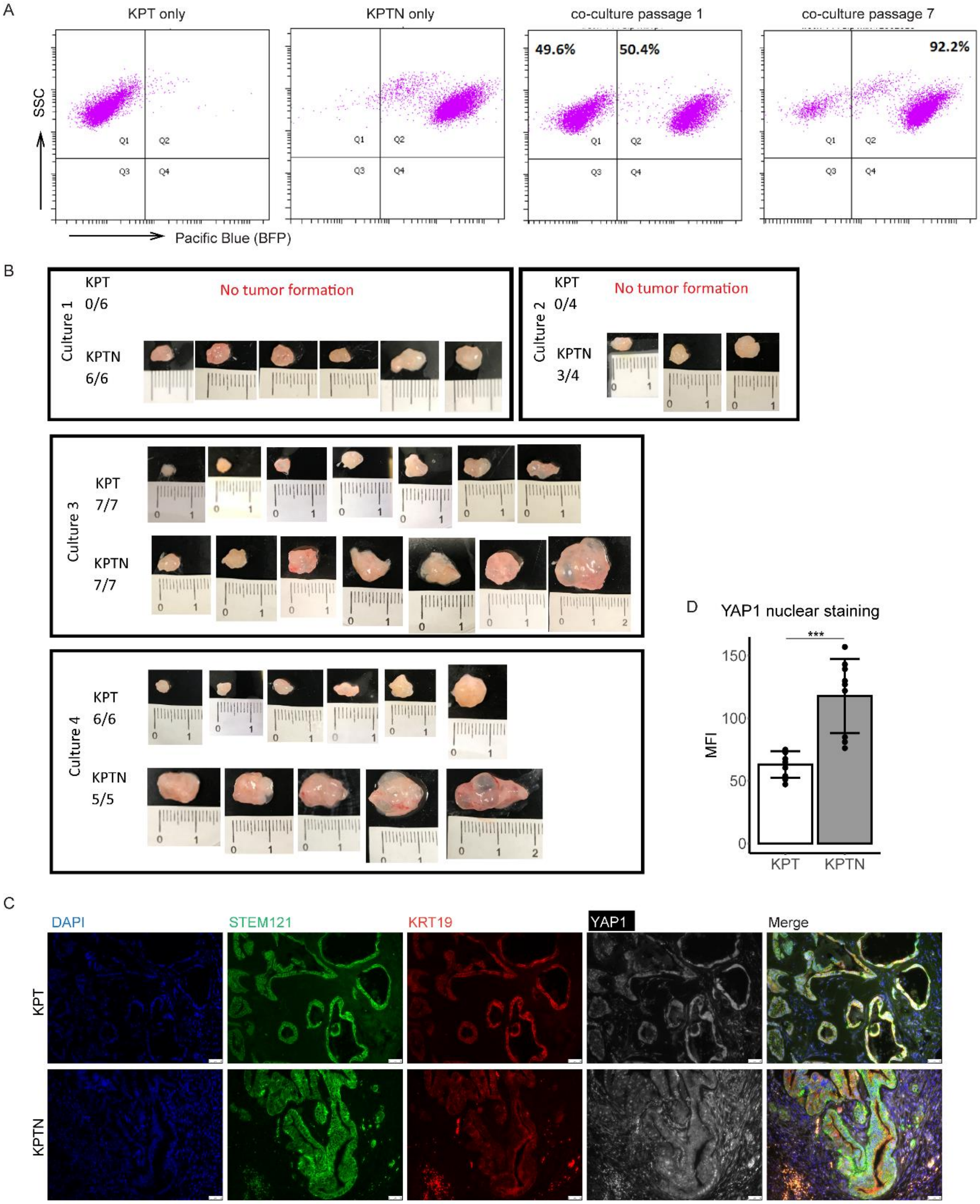
A
KPT only
KPTN only
co-culture passage 1
co-culture passage 7
49.6%
50.4%
92.2%
SSC
Pacific Blue (BFP)
Q1
Q2
Q3
Q4
B
Culture 1
KPT 0/6
No tumor formation
KPTN 6/6
Culture 2
KPT 0/4
No tumor formation
KPTN 3/4
Culture 3
KPT 7/7
KPTN 7/7
Culture 4
KPT 6/6
KPTN 5/5
D
YAP1 nuclear staining
***
MFI
150
100
50
0
KPT
KPTN
C
DAPI
STEM121
KRT19
YAP1
Merge
KPT
KPTN

Figure S3. Loss of *NF2* results in growth advantage of engineered acinar cells *in vitro* and *in vivo*. A. Flow cytometry analysis of BFP signal in KPT culture, KPTN culture or their coculture at 1:1 ratio. **B.** Photos of all xenograft tumors harvested from NSG mice transplanted with KPT or KPTN organoids. **C.** Low magnification images of the immunofluorescence staining as shown in **Figure 2E**. Cell nuclei was counter stained with DAPI. Scale bar = 50 µm. **D.** Quantification of YAP1 nuclei staining from 3 sections with 3 random fields of view for each. Data was analyzed by two-tailed Student's t test. Error bar represents standard deviation. *** indicates p-value < 0.001.

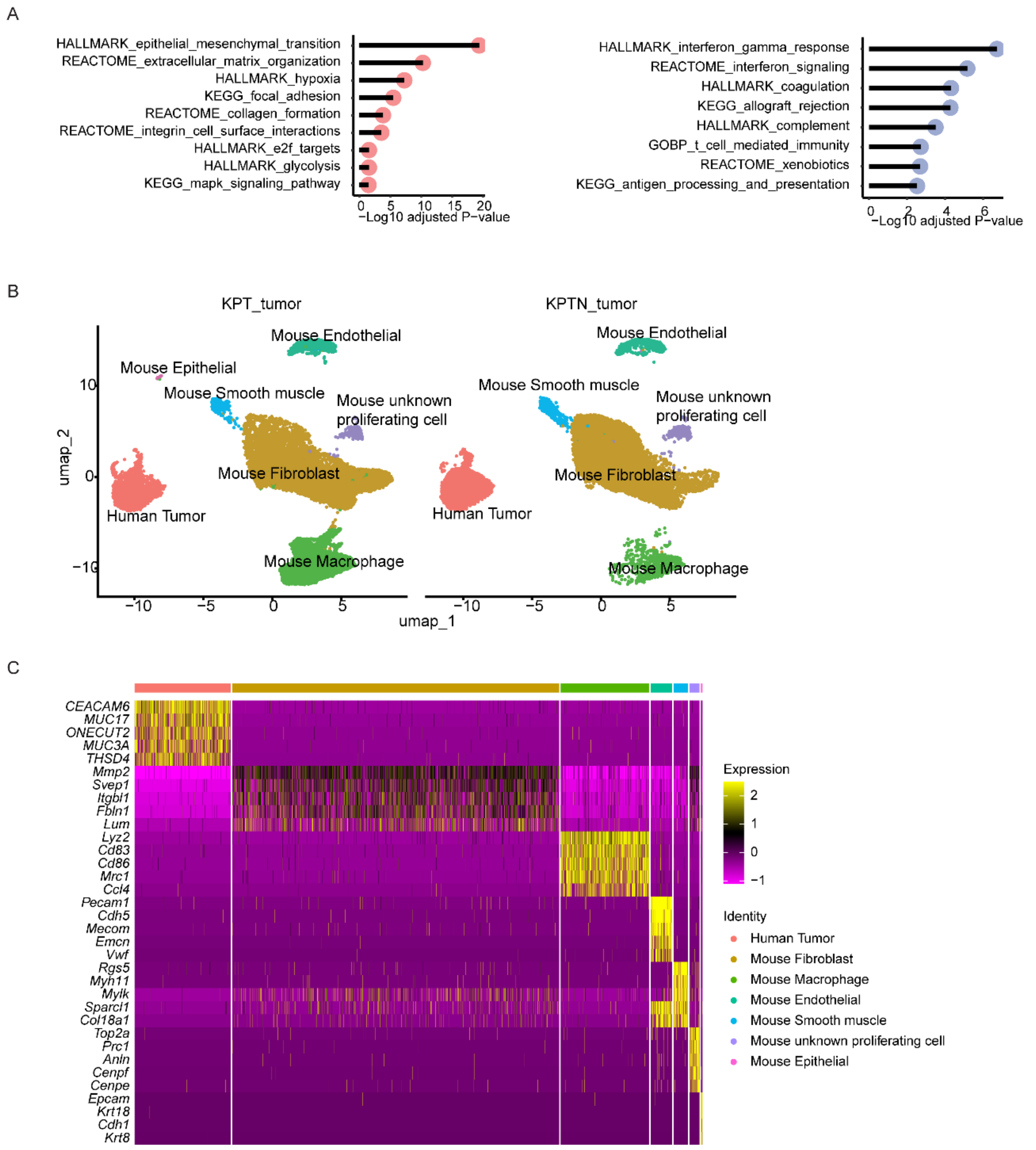
A
HALLMARK_epithelial_mesenchymal_transition
REACTOME_extracellular_matrix_organization
HALLMARK_hypoxia
KEGG_focal_adhesion
REACTOME_collagen_formation
REACTOME_integrin_cell_surface_interactions
HALLMARK_e2f_targets
HALLMARK_glycolysis
KEGG_mapk_signaling_pathway
−Log10 adjusted P−value
HALLMARK_interferon_gamma_response
REACTOME_interferon_signaling
HALLMARK_coagulation
KEGG_allograft_rejection
HALLMARK_complement
GOBP_t_cell_mediated_immunity
REACTOME_xenobiotics
KEGG_antigen_processing_and_presentation
−Log10 adjusted P−value
B
KPT_tumor
KPTN_tumor
Mouse Endothelial
Mouse Epithelial
Mouse Smooth muscle
Mouse unknown proliferating cell
Mouse Fibroblast
Human Tumor
Mouse Macrophage
umap_1
umap_2
C
CEACAM6
MUC17
ONECUT2
MUC3A
THSD4
Mmp2
Svep1
Itgbl1
Fbln1
Lum
Lyz2
Cd83
Cd86
Mrc1
Ccl4
Pecam1
Cdh5
Mecom
Emcn
Vwf
Rgs5
Myh11
Mylk
Sparcl1
Col18a1
Top2a
Prc1
Anln
Cenpf
Cenpe
Epcam
Krt18
Cdh1
Krt8
Expression
Identity
Human Tumor
Mouse Fibroblast
Mouse Macrophage
Mouse Endothelial
Mouse Smooth muscle
Mouse unknown proliferating cell
Mouse Epithelial

Figure S4. Transcriptomic analysis of KPT versus KPTN tumors. A. Overrepresentation analysis for upregulated (in red) and downregulated genes (in blue) in KPTN tumor as shown in **Figure 3A**. A significant enrichment at pathway level was considered with multiple-test adjusted p-value < 0.05. **B.** Annotation of all cell populations present in KPT and KPTN-derived tumor tissues in single cell RNA seq analysis. **C.** Expression heatmap of top feature genes in each cell populations as shown in **B**.

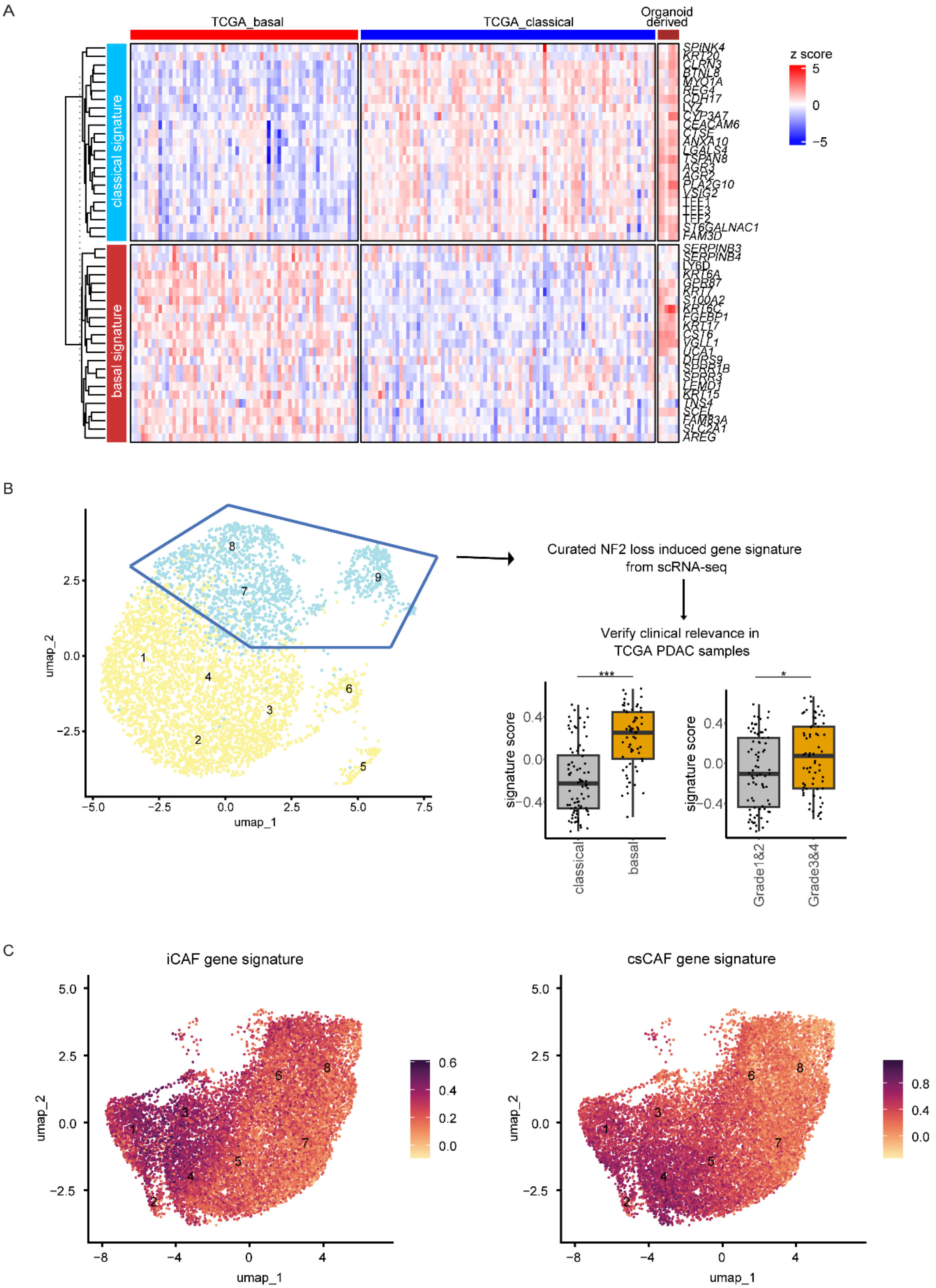

A
TCGA_basal
TCGA_classical
Organoid derived
z score
5
0
−5
classical signature
basal signature
SPINK4
KRT20
CLRN3
BTNL8
MYO1A
REG4
CDH17
LYZ
CYP3A7
CEACAM6
CTSE
ANXA10
LGALS4
TSPAN8
AGR3
AGR2
PLA2G10
VSIG2
TFF1
TFF3
TFF2
ST6GALNAC1
FAM3D
SERPINB3
SERPINB4
LY6D
KRT6A
GPR87
KRT7
S100A2
KRT6C
FGFBP1
KRT17
CST6
VGLL1
UCA1
DHRS9
SPRR1B
SPRR3
LEMD1
KRT15
TNS4
SCEL
FAM83A
SLC2A1
AREG
B
umap_2
umap_1
Curated NF2 loss induced gene signature from scRNA-seq
Verify clinical relevance in TCGA PDAC samples
signature score
***
*
classical
basal
Grade1&2
Grade3&4
C
iCAF gene signature
csCAF gene signature
umap_2
umap_1

Figure S5. Comparison of engineered acinar organoid-derived tumors with TCGA PDAC samples. A. Expression heatmap of classic and basal PDAC subtype gene signatures in TCGA PDAC samples as well as KPT and KPTN tumor samples generated in the present study. **B.** Expression of *NF2* induced malignancy-associated genes curated from single cell RNA seq data in TCGA PDAC samples. TCGA PDAC samples were grouped by subtype or grade. The middle line of box represents the median value, the bounds of box represent the Interquartile Range, and the whiskers extend to 1.5 × Interquartile Range. Statistical analysis was performed by two-tailed Student's t test. *, *** indicate p-value < 0.05 and 0.001, respectively, between 2 groups. **C.** Expression of indicated gene modules in all the populations as shown in **Figure 4A**.

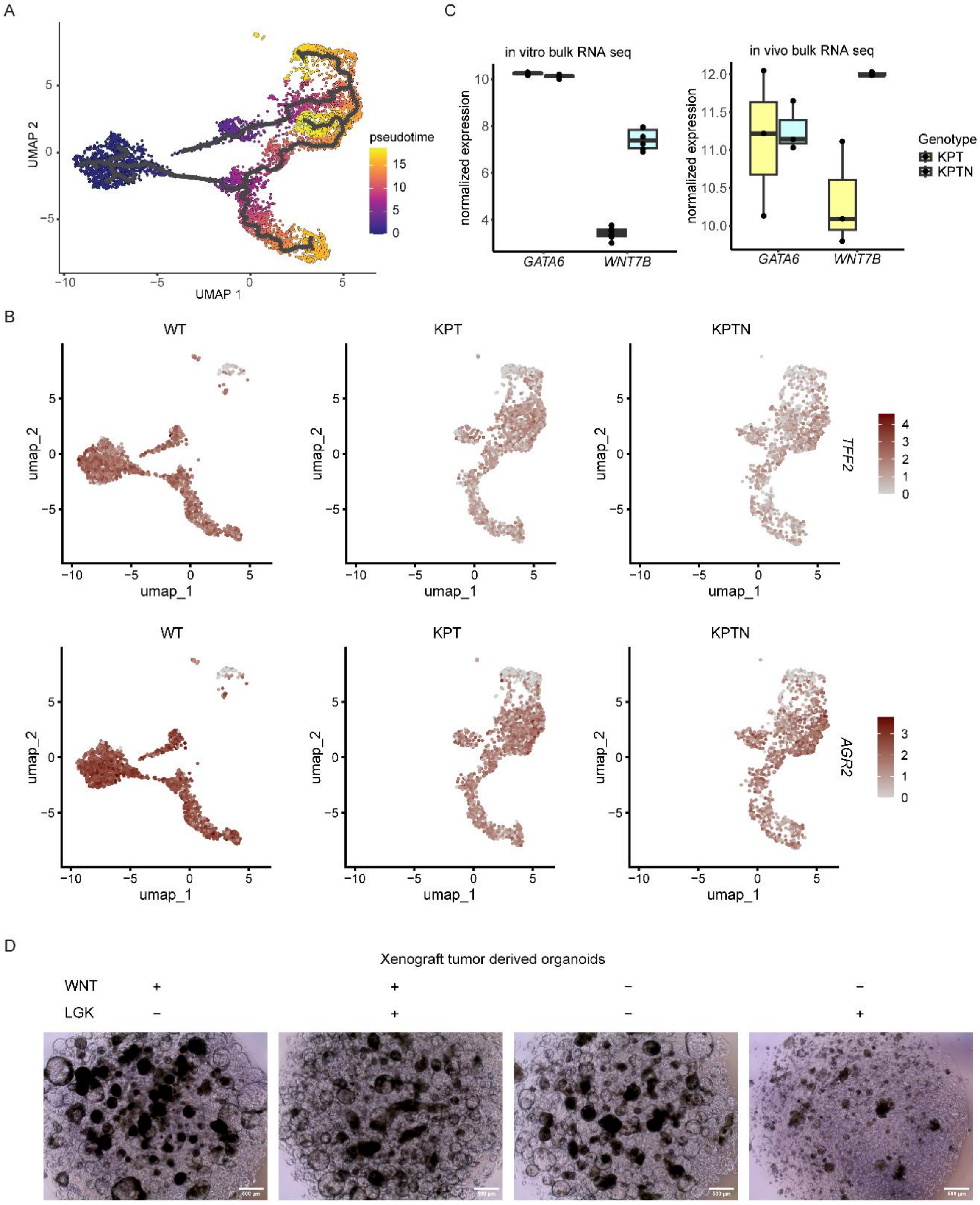
A
C
B
D
pseudotime
UMAP 1
UMAP 2
in vitro bulk RNA seq
in vivo bulk RNA seq
normalized expression
GATA6
WNT7B
Genotype
KPT
KPTN
WT
KPT
KPTN
umap_1
umap_2
TFF2
AGR2
Xenograft tumor derived organoids
WNT
LGK

Figure S6. Transcriptomic analysis of engineered acinar organoid cultures. A. Pseudo time trajectory analysis of all cell populations shown in **Figure 5C**. **B.** Expression level of indicated genes in all the cells populations shown in **Figure 5C**. **C.** Log2 transformed normalized gene expression of *WNT7B* and *GATA6* in bulk RNA seq analysis of KPT and KPTN *in vitro* cultures (left, n = 6 each) and xenograft tumors (right, n = 3 each). The middle line of box represents the median value, the bounds of box represent the Interquartile Range, and the whiskers extend to 1.5 × Interquartile Range. **D.** Representative images of tumor-derived organoids incubated with or without WNT3A supplement or LGK974 treatment (100 nM) from 4 biological replicates. Scale bar = 500 μm.

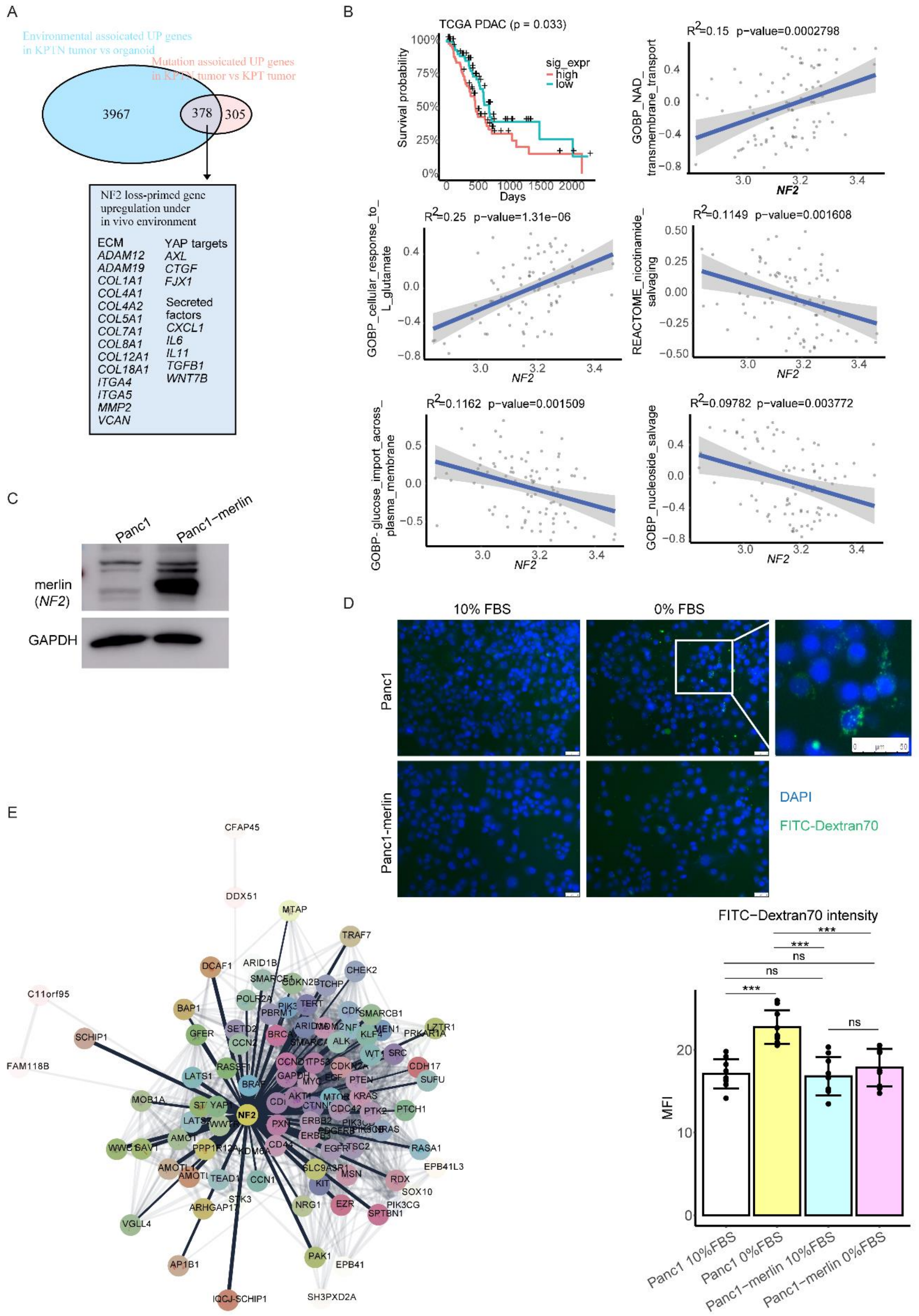
A
Environmental assoicated UP genes
in KP1N tumor vs organoid
Mutation assoicated UP genes
in KP1N tumor vs KPT tumor
3967
378
305
NF2 loss-primed gene
upregulation under
in vivo environment
ECM
ADAM12
ADAM19
COL1A1
COL4A1
COL4A2
COL5A1
COL7A1
COL8A1
COL12A1
COL18A1
ITGA4
ITGA5
MMP2
VCAN
YAP targets
AXL
CTGF
FJX1
Secreted factors
CXCL1
IL6
IL11
TGFB1
WNT7B
B
TCGA PDAC (p = 0.033)
Survival probability
Days
sig_expr
high
low
R2=0.15 p-value=0.0002798
GOBP_NAD_transmembrane_transport
R2=0.25 p-value=1.31e-06
GOBP_cellular_response_to_L_glutamate
R2=0.1149 p-value=0.001608
REACTOME_nicotinamide_salvaging
R2=0.1162 p-value=0.001509
GOBP_glucose_import_across_plasma_membrane
R2=0.09782 p-value=0.003772
GOBP_nucleoside_salvage
NF2
C
Panc1
Panc1−merlin
merlin (NF2)
GAPDH
D
10% FBS
0% FBS
Panc1
Panc1-merlin
DAPI
FITC-Dextran70
FITC−Dextran70 intensity
MFI
***
***
ns
ns
***
ns
Panc1 10%FBS
Panc1 0%FBS
Panc1−merlin 10%FBS
Panc1−merlin 0%FBS
E
CFAP45
DDX51
MTAP
TRAF7
C11orf95
FAM118B
SCHIP1
NF2
EPB41L3
SOX10
PIK3CG
SPTBN1
EPB41
SH3PXD2A
PAK1
AP1B1
IQCJ-SCHIP1
VGLL4
ARHGAP17
LZTR1
PRKAR1A
CDH17
SUFU
PTCH1
RASA1

Figure S7. ***NF2*** **inactivation promotes cell survival under nutrient deprivation via enhanced macropinocytosis. A.** Comparison of environmentally and genetically induced upregulated genes in KPTN tumors. Environmentally induced upregulated genes were curated from the comparison of KPTN tumors vs organoids (shown in **Figure 7A**). *NF2* loss induced upregulated genes was curated from the comparison of KPTN tumors vs KPT tumors (shown in **Figure 3A**). **B.** Top left: KM plot of TCGA PDAC patient survival separated by median expression score of starvation associated genes as described in **Figure 7A-B**. Top right, middle and bottom: Correlation of indicated pathways with *NF2* expression level in TCGA PDAC samples. **C.** Western blot of merlin protein expression in Panc1 cells transfected with pcDNA3-merlin plasmid or an empty vector. **D.** Representative images and quantification of FITC-Dextran70 signal in Panc1 cells cultured in 10% FBS or 0% FBS with or without merlin overexpression. Quantification was performed from 3 sections with 3 random fields of view for each. Data was analyzed by two-tailed Student's t test. Error bar represents standard deviation. ns and *** indicate no significance and p-value < 0.001, respectively. **E.** Protein-protein interaction network of *NF2* in STRING database.

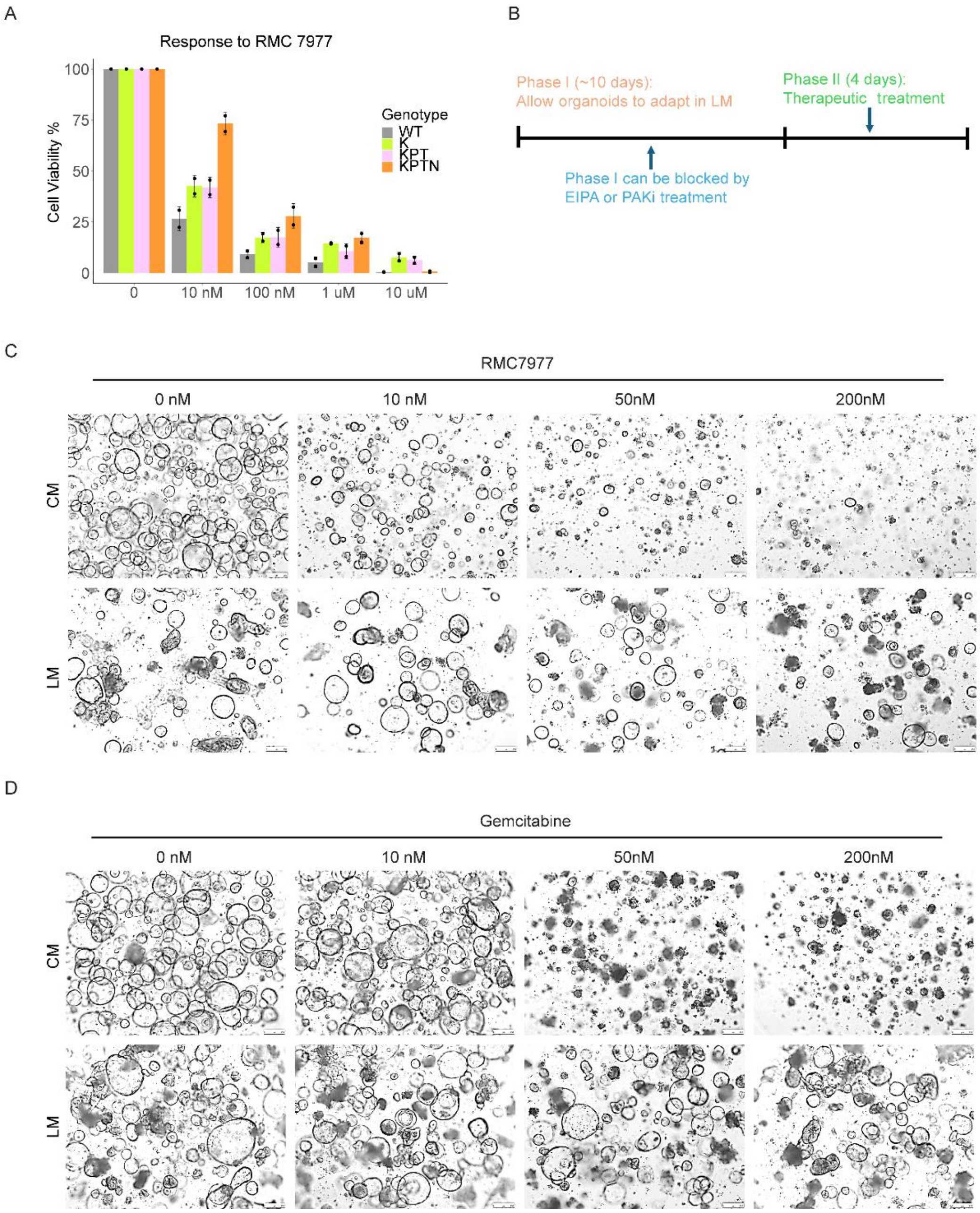
A
Response to RMC 7977
Cell Viability %
100
75
50
25
0
0
10 nM
100 nM
1 uM
10 uM
Genotype
WT
K
KPT
KPTN
B
Phase I (~10 days):
Allow organoids to adapt in LM
Phase II (4 days):
Therapeutic treatment
Phase I can be blocked by
EIPA or PAKi treatment
C
RMC7977
0 nM
10 nM
50nM
200nM
CM
LM
D
Gemcitabine
0 nM
10 nM
50nM
200nM
CM
LM

Figure S8. ***NF2* loss and nutrient starvation cooperate to induce therapeutic resistance. A.** Response to RMC 7977 from acinar 3D cultures harboring different genetic backgrounds (n = 2 for each genotype). K: *KRAS* mutation, KPT: *KRAS*, *CDKN2A/p16*, *TP53* triple mutation, KPTN: *KRAS*, *CDKN2A/p16*, *TP53*, *NF2* quadruple mutation. **B.** Schematic illustration of experimental workflow for **Figure 9A-F**. **C.** Representative images of KPTN organoids cultured in CM or LM and treated with RMC 7977 at indicated doses or vehicle control from 4 independent experiments. Scale bar = 250 µm. **D.** Representative images of KPTN organoids cultured in CM or LM and treated with Gemcitabine at indicated doses or vehicle control from 4 independent experiments. Scale bar = 250 µm.

Supplementary Table List

Supplementary Table 1. Organ donor information of the pancreatic tissues used in this study.

Supplementary Table 2. sgRNA sequences of the CRISPR knockout library.

Supplementary Table 3. Enrichment analysis summary at gene level for the *in vivo* and *in vitro* CRISPR screen.

Supplementary Table 4. Enriched target genes from CRISPR screen described in **Figure 1D** and the core signaling pathways in human pancreatic cancer in which the indicated target genes are involved.

Supplementary Table 5. List of DEGs from bulk RNA seq analysis described in **Figure 3A**, **5A** and **7A**.

Supplementary Table 6. Top 20 marker genes for each cell population from scRNA seq analysis described in **Figure 3B**, **4A** and **5C**.

Supplementary Table 7. Other curated gene signatures from the present or previous studies.
