# Supplementary figures and images for: NF2 loss malignantly transforms human pancreatic acinar cells and enhances cell fitness under environmental stress
Source: J Clin Invest. 2026 Jan 2;136(1):e194395. doi: 10.1172/JCI194395 (PMC12721884; doi:10.1172/JCI194395)

Full unedited gel for **Figure 2B**

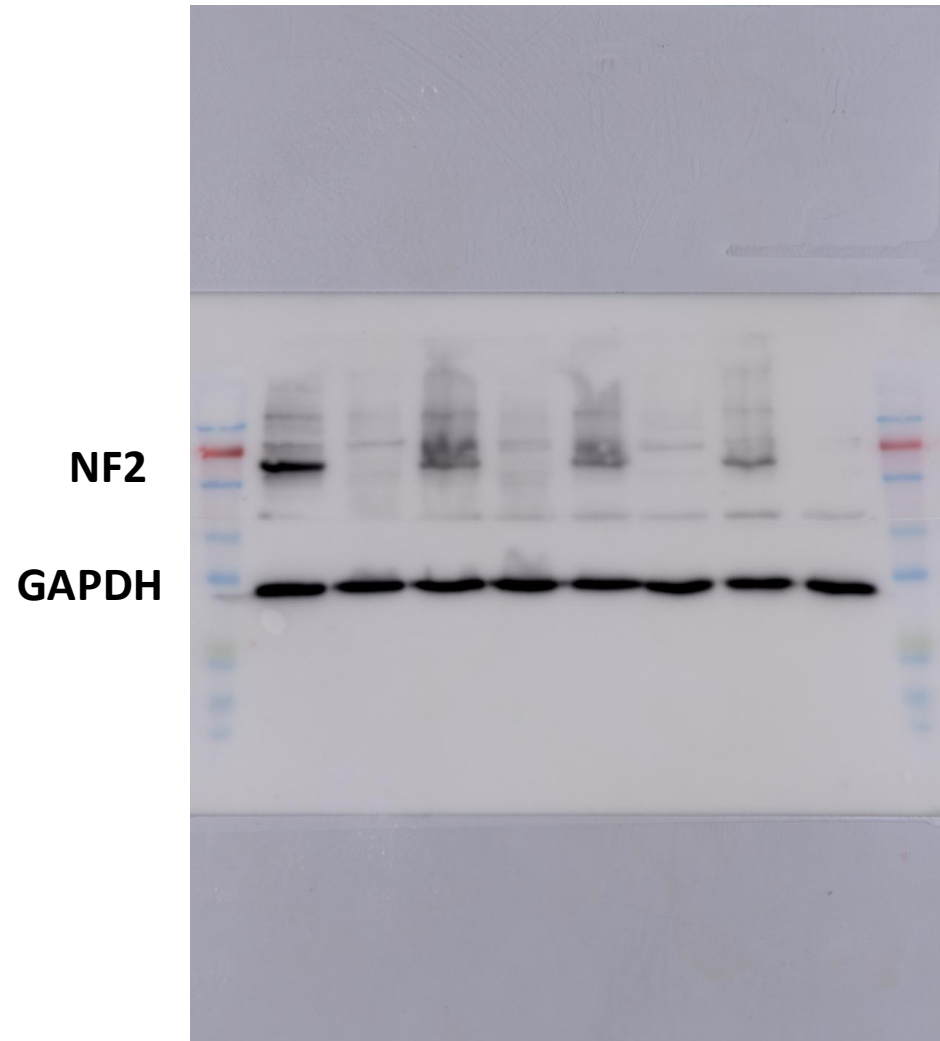

Full unedited gel for **Figure S1C**

**p53**

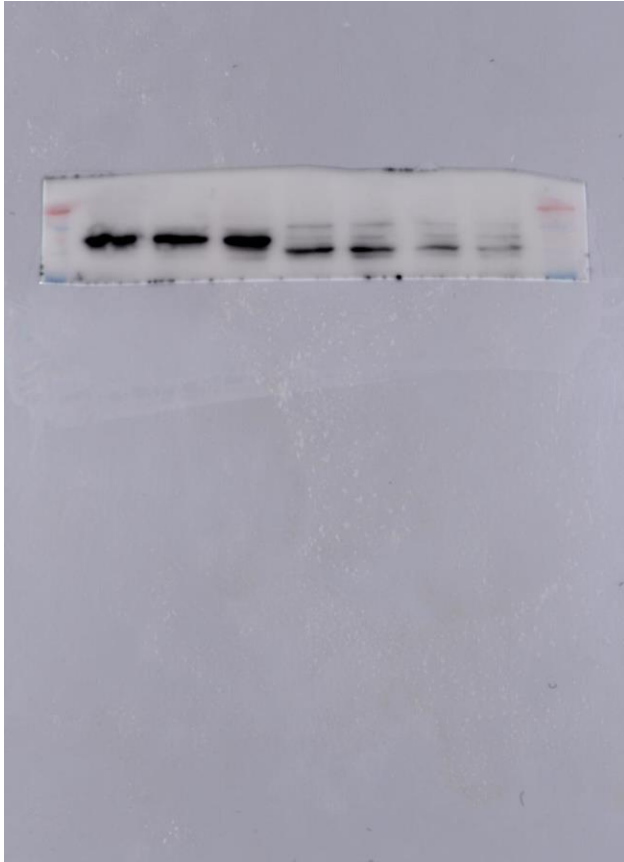

**KRAS**

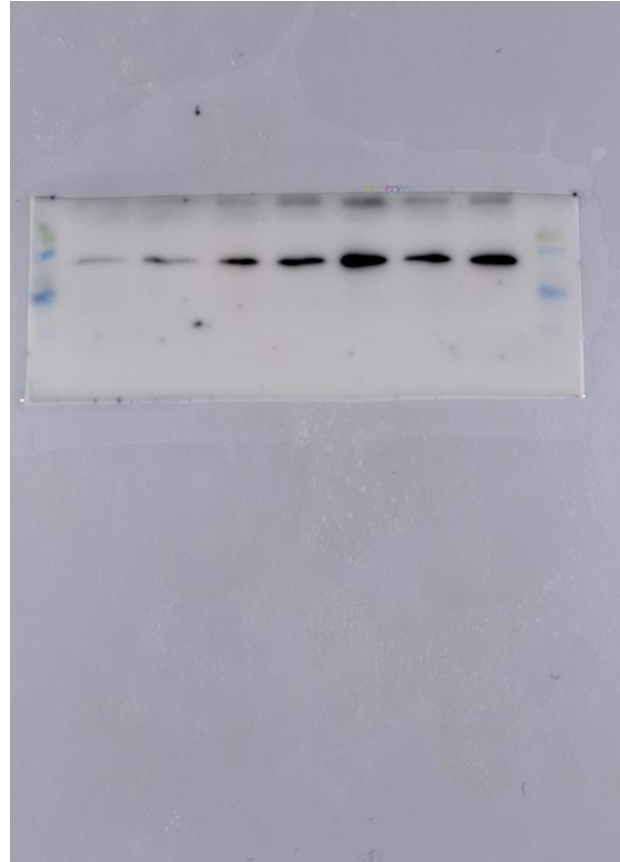

**GAPDH**

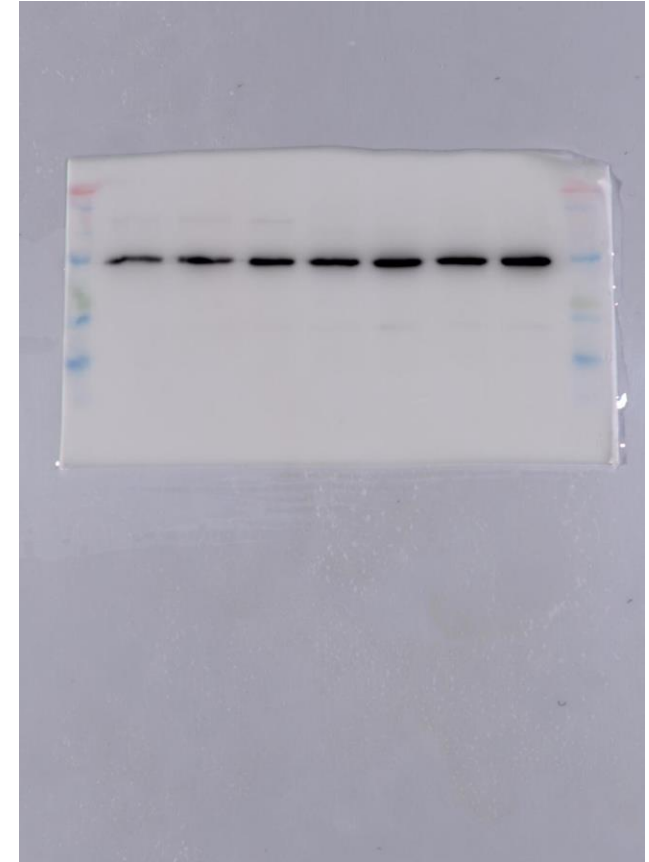

Supplement: Unedited blot and gel images [file jci-136-194395-s119.pdf]
